# Urinary EGF and MCP-1 and risk of CKD after cardiac surgery

**DOI:** 10.1172/jci.insight.147464

**Published:** 2021-06-08

**Authors:** Steven Menez, Wenjun Ju, Rajasree Menon, Dennis G. Moledina, Heather Thiessen Philbrook, Eric McArthur, Yaqi Jia, Wassim Obeid, Sherry G. Mansour, Jay L. Koyner, Michael G. Shlipak, Steven G. Coca, Amit X. Garg, Andrew S. Bomback, John A. Kellum, Matthias Kretzler, Chirag R. Parikh, for the Translational Research Investigating Biomarker Endpoints in AKI (TRIBE-AKI) Consortium and the Kidney Precision Medicine Project

**Affiliations:** 1 Division of Nephrology, Department of Medicine, Johns Hopkins University School of Medicine, Baltimore, Maryland, USA.; 2 Division of Nephrology, Department of Medicine, and Department of Computational Medicine and Bioinformatics, University of Michigan, Ann Arbor, Michigan, USA.; 3 Section of Nephrology and; 4 Clinical and Translational Research Accelerator, Department of Internal Medicine, Yale University School of Medicine, New Haven, Connecticut, USA.; 5 ICES, Ontario, Canada.; 6 Section of Nephrology, Department of Medicine, University of Chicago, Chicago, Illinois, USA.; 7 Kidney Health Research Collaborative and Division of General Internal Medicine, San Francisco Veterans Affairs Medical Center, University of California San Francisco, San Francisco, California, USA.; 8 Division of Nephrology, Department of Medicine, Icahn School of Medicine at Mount Sinai, New York, New York, USA.; 9 Division of Nephrology, Department of Medicine, and; 10 Department of Epidemiology and Biostatistics, Schulich School of Medicine & Dentistry, Western University, London, Ontario, Canada.; 11 Division of Nephrology, Department of Medicine, Columbia University Vagelos College of Physicians and Surgeons, New York, New York, USA.; 12 The Center for Critical Care Nephrology, Department of Critical Care Medicine, University of Pittsburgh, Pittsburgh, Pennsylvania, USA.; 13 The TRIBE-AKI Consortium and the Kidney Precision Medicine Project are detailed in Supplemental Acknowledgments.

**Keywords:** Nephrology, Cardiovascular disease, Chronic kidney disease, Molecular genetics

## Abstract

**BACKGROUND:**

Assessment of chronic kidney disease (CKD) risk after acute kidney injury (AKI) is based on limited markers primarily reflecting glomerular function. We evaluated markers of cell integrity (EGF) and inflammation (monocyte chemoattractant protein-1, MCP-1) for predicting long-term kidney outcomes after cardiac surgery.

**METHODS:**

We measured EGF and MCP-1 in postoperative urine samples from 865 adults who underwent cardiac surgery at 2 sites in Canada and the United States and assessed EGF and MCP-1’s associations with the composite outcome of CKD incidence or progression. We used single-cell RNA-Seq (scRNA-Seq) of AKI patient biopsies to perform transcriptomic analysis of programs corregulated with the associated genes.

**RESULTS:**

Over a median (IQR) follow-up of 5.8 (4.2–7.1) years, 266 (30.8%) patients developed the composite CKD outcome. Postoperatively, higher levels of urinary EGF were protective and higher levels of MCP-1 were associated with the composite CKD outcome (adjusted HR 0.83, 95% CI 0.73–0.95 and 1.10, 95% CI 1.00–1.21, respectively). Intrarenal scRNA-Seq transcriptomes in patients with AKI-defined cell populations revealed concordant changes in EGF and MCP-1 levels and underlying molecular processes associated with loss of *EGF* expression and gain of *CCL2* (encoding MCP-1) expression.

**CONCLUSION:**

Urinary EGF and MCP-1 were each independently associated with CKD after cardiac surgery. These markers may serve as noninvasive indicators of tubular damage, supported by tissue transcriptomes, and provide an opportunity for novel interventions in cardiac surgery.

**TRIAL REGISTRATION:**

ClinicalTrials.gov NCT00774137.

**FUNDING:**

The NIH funded the TRIBE-AKI Consortium and Kidney Precision Medicine Project. Yale O’Brien Kidney Center, American Heart Association, Patterson Trust Fund, Dr. Adam Linton Chair in Kidney Health Analytics, Canadian Institutes of Health Research, ICES, Ontario Ministry of Health and Long-Term Care, Academic Medical Organization of Southwestern Ontario, Schulich School of Medicine & Dentistry, Western University, Lawson Health Research Institute, Chan Zuckerberg Initiative Human Cell Atlas Kidney Seed Network.

## Graphical abstract

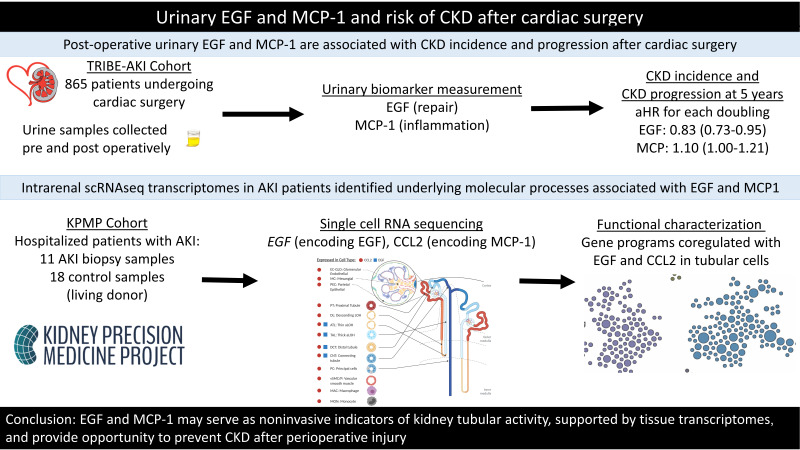

## Introduction

Globally, over 1 million patients undergo cardiac surgery every year (1, 2). Although perioperative mortality rate is low, many survivors develop long-term chronic kidney disease (CKD) as a late complication (3–5). Thus, cardiac surgery indirectly contributes to CKD burden, especially because many patients have preexisting risk factors such as diabetes, hypertension, and older age (6, 7). Cardiac surgery also serves as an ideal setting to study both short-term and long-term kidney outcomes, given the predictable and clear timing of injury.

The clinical detection of acute kidney injury (AKI) and CKD is currently based on a set of markers that primarily reflect glomerular function and structure (serum creatinine and proteinuria). The narrow definition of kidney function reflected by the selection of these clinical markers may limit the ability to detect early stages of impending AKI or CKD. Current efforts have been focused on more completely capturing kidney function by testing biomarkers as surrogates for organ function, injury, inflammation, and repair. Biomarkers have been increasingly studied across the spectrum of kidney disease, from AKI to CKD, to detect early or subclinical injury. In AKI, individuals who do not experience a rise in serum creatinine but have elevated levels of kidney injury biomarkers (termed *subclinical AKI*) compared with those with normal biomarker levels have significantly worse outcomes, including a higher risk of death and receipt of kidney replacement therapy (8–12). Furthermore, kidney biopsies in patients with subclinical AKI reveal structural evidence of injury that will lead to CKD if the injured areas are not repaired appropriately (8).

As with AKI, urinary biomarkers of chronic kidney damage have demonstrated strong associations with disease progression across various clinical settings. Lower levels of urinary EGF, an epithelial integrity and repair biomarker, and higher levels of urinary monocyte chemoattractant protein-1 (MCP-1), an inflammatory biomarker, are associated with greater interstitial fibrosis and tubular atrophy (13, 14). Higher levels of EGF are associated with better therapeutic response and disease remission in various glomerular diseases (13, 15). In a single-center study of urinary cytokines in the setting of ischemic AKI, relatively higher urinary EGF was associated with kidney function recovery at 7 days but also with mortality at 3 months based on receiver operating characteristic curve (16).

Furthermore, both urinary EGF and MCP-1 have been studied extensively in the setting of diabetic kidney disease, and both lower urinary EGF and higher MCP-1 are significantly associated with greater disease progression (17, 18). In addition to the importance of urinary EGF and MCP-1 measured independently, a lower EGF/MCP-1 ratio predicts kidney function decline in diabetes mellitus, IgA nephropathy, and various other glomerular diseases (13, 19).

While most AKI following cardiac surgery is mild and resolves spontaneously, the risk of CKD postsurgery remains high. We need better biomarkers, therefore, to detect kidney injury that leads to CKD. To our knowledge, the roles of these biomarkers of injury, inflammation, or repair in settings of AKI transitioning toward CKD have not been studied in a large cohort. As AKI is common after cardiac surgery, we used this setting to test the ability of these markers of kidney function and failure to predict CKD progression, independent of clinical AKI, and to link the urinary biomarkers to specific intrarenal cell populations and to the molecular programs that regulate expression of these biomarkers. Therefore, in this study, we first examined the associations of urinary EGF, urinary MCP-1, and the EGF/MCP-1 ratio with a composite outcome of CKD incidence or progression in patients undergoing cardiac surgery. In addition, using single-cell RNA sequencing (scRNA-Seq) data sets from protocol renal biopsies of patients with AKI, we defined cell populations with differential expression of *EGF* and *CCL2* (the gene encoding MCP-1 protein) transcripts in patients with AKI, compared with living donor (LD) samples as a control. Finally, to define the upstream regulatory events associated with the differential intrarenal expression of the prognostic biomarkers, we compared, on an individual-cell level, the molecular programs linked to loss of *EGF* and induction of *CCL2*. We hypothesized that lower urinary EGF levels and higher urinary MCP-1 levels in the immediate postoperative period would associate with a higher risk of CKD years later. We additionally hypothesized that *EGF* expression would be lower in the setting of AKI compared with LD, while *CCL2* expression would be higher in AKI compared with LD, with *EGF* and *CCL2* expression both highest in the distal tubule.

## Results

### Study population.

**Figure 1:**
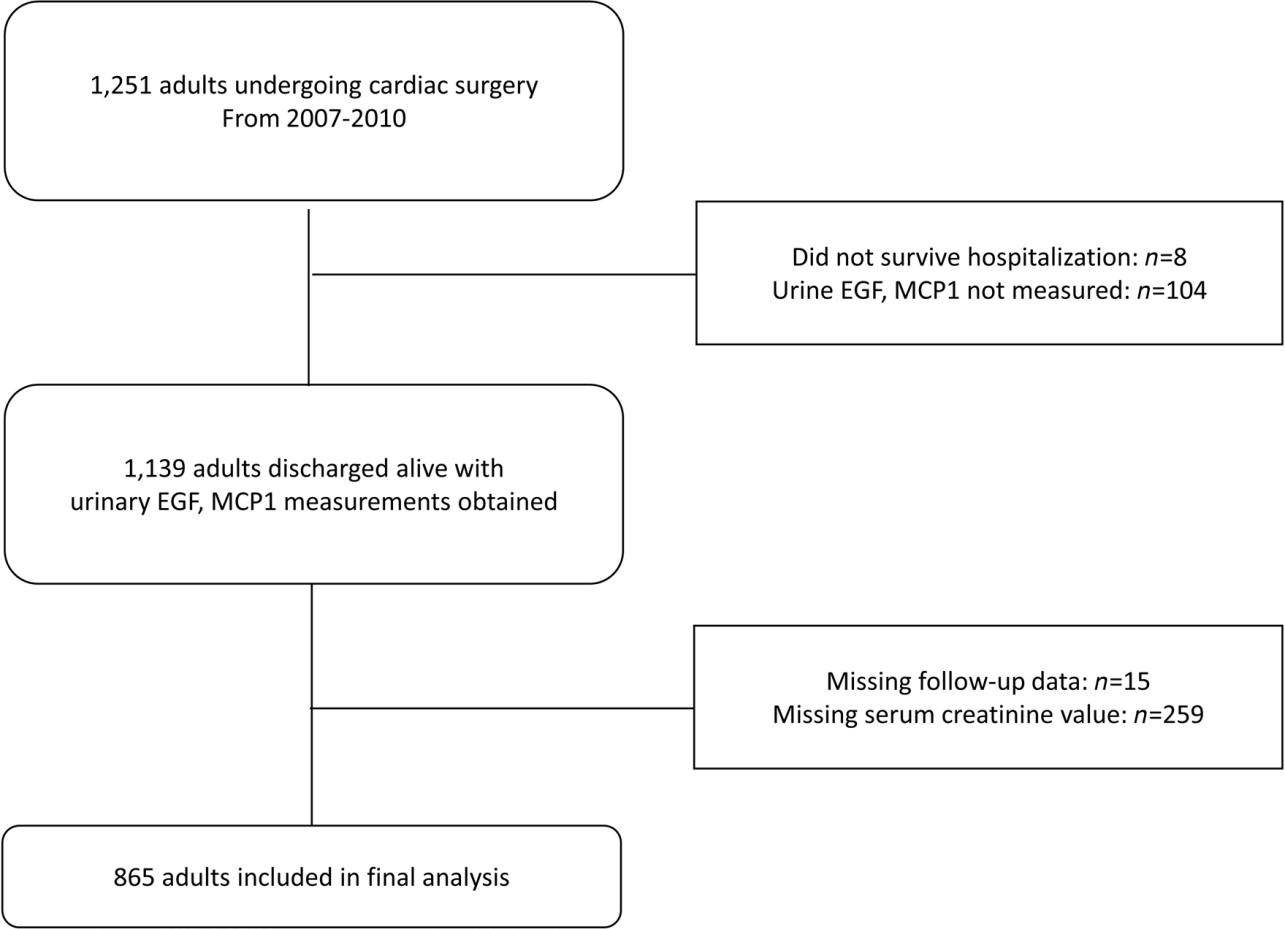
Flow diagram of TRIBE-AKI Study. Among patients in the 2 study sites (Ontario, Canada, and New Haven, Connecticut, USA) undergoing cardiac surgery, a total of 1139 were discharged alive from the hospital, with a total of 865 patients with follow-up data and creatinine available for this study.

**Figure 2:**
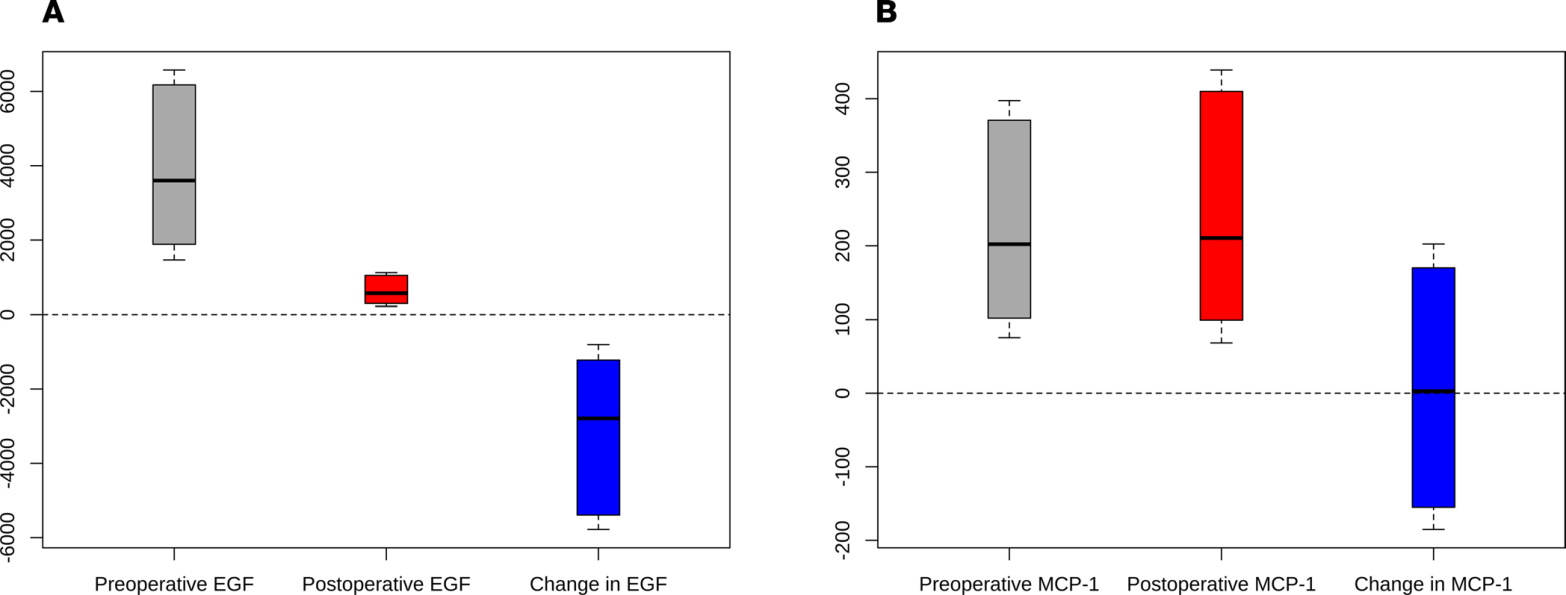
Effect of cardiac surgery on urinary biomarker levels. Changes in preoperative to postoperative urinary EGF (**A**) and monocyte chemoattractant protein-1 (MCP-1) (**B**) in pg/mL. The horizontal lines represent the median and bounds of box-and-whisker plots representing the 25th and 75th percentiles, with upper and lower extremes represented by whiskers.

Of the 1251 patients who underwent cardiac surgery 2007–2010 at the 2 Translational Research Investigating Biomarker Endpoints in AKI (TRIBE-AKI) study sites, 865 (69%) had biomarker measurements and at least 1 serum creatinine measurement available upon follow-up and composed our final analytic population (Figure 1). Table 1 describes baseline characteristics for participants by postoperative urinary EGF tertiles. The mean patient age at the time of surgery was 71.8 (9.0) years, and 615 (71%) patients were male. The mean baseline eGFR was 69.5 (18.1) mL/min/1.73 m^2^, and the median urine albumin/creatinine ratio was 64.0 mg/g (187 mg/g). Patients in EGF tertiles 2 and 3 had relatively higher baseline eGFRs compared with those in tertile 1. The median (IQR) difference in urinary EGF level was 2710 (1101 to 5294) pg/mL, or 82.4% (64.4% to 91.9%), from the median preoperative to median postoperative value (Figure 2A). Supplemental Table 1 (supplemental material available online with this article; https://doi.org/10.1172/jci.insight.147464DS1) describes baseline characteristics by postoperative urinary MCP-1 tertile. Patients in MCP-1 tertiles 2 and 3 had relatively lower baseline eGFR compared with those in tertile 1. The median (IQR) difference between the preoperative and postoperative urinary MCP-1 level was 3.3 (–161 to 185) pg/mL, or 2.7% (–56.3% to 156.4%) (Figure 2B). The correlation between preoperative and postoperative EGF was 0.28, whereas the correlation between preoperative and postoperative MCP-1 was 0.19.

Over a median follow-up of 5.8 (IQR 4.2–7.1) years, 266 (30.7%) patients developed the composite CKD outcome (primary outcome), with an event rate of 55.4 (49.2–62.5) per 1000 person-years of follow-up (Supplemental Table 2). Two hundred seventy-one (31.3%) patients experienced clinical AKI during hospitalization, and 105 (38.7%) of them developed the primary outcome. Of the remaining 594 patients who did not experience clinical AKI during hospitalization, 161 (27.1%) developed the primary outcome.

### Association of postoperative biomarkers with the composite CKD outcome.

Each 2-fold higher level of postoperative urinary EGF was associated with a decreased risk of the composite CKD outcome after adjusting for urine creatinine (HR 0.78, 95% CI 0.69–0.89) (Table 2). After full adjustment for potential confounders, including demographic characteristics, medical comorbidities, and surgical factors, the decreased risk of the composite CKD outcome persisted (adjusted HR [aHR] 0.83, 95% CI 0.73–0.95). In the categorical analysis, patients in tertile 3 of urinary EGF level had a decreased risk of the composite CKD outcome compared with those in tertile 1, although this was not statistically significant (aHR 0.68, 95% CI 0.43–1.07) (Supplemental Table 3).

Each 2-fold higher level of postoperative urinary MCP-1 was associated with an increased risk of the composite CKD outcome after adjustment for urine creatinine (HR 1.12, 95% CI 1.04–1.21). This association persisted after full adjustment (aHR 1.10, 95% CI 1.00–1.21). Patients in tertile 3 of urinary MCP-1 level had an increased risk of the composite CKD outcome compared with those in tertile 1, which approached statistical significance (aHR 1.48, 95% CI 0.99–2.22).

Each 2-fold higher level of the postoperative EGF/MCP-1 ratio was associated with a decreased risk of the composite CKD outcome after adjustment for urine creatinine (HR 0.86, 95% CI 0.81–0.92). This remained significant after full adjustment for potential confounders (aHR 0.86, 95% CI 0.79–0.94). In categorical analysis by EGF/MCP-1 ratio tertile, after full adjustment, patients in tertile 3 of EGF/MCP-1 level had a significantly decreased risk of the composite CKD outcome compared with those in tertile 1 (aHR 0.50, 95% CI 0.33–0.74).

### Association of preoperative biomarkers with the composite CKD outcome.

We also evaluated the association between preoperative biomarkers with the composite CKD outcome (Supplemental Table 4). Higher levels of preoperative urinary EGF were not associated with a significant risk of the composite CKD outcome (aHR 0.99, 95% CI 0.85–1.15). However, higher levels of preoperative urinary MCP-1 were associated with increased risk of the composite CKD outcome (aHR 1.23, 95% CI 1.10–1.38). Conversely, higher levels of preoperative EGF/MCP-1 were associated with a decreased risk (aHR 0.79, 95% CI 0.71–0.89).

We next evaluated the association of postoperative urinary EGF, urinary MCP-1, and their ratio with the composite CKD outcome, adjusting for their preoperative values (Supplemental Table 5). We observed largely similar results, with each 2-fold higher level of the urinary EGF and EGF/MCP-1 associated with lower risk of the primary outcome (aHR 0.84 [95% CI 0.73–0.96] and 0.91 [95% CI 0.83–0.99], respectively). However, the association with postoperative MCP-1 was attenuated after adjusting for preoperative levels (aHR 1.08, 95% CI 0.98–1.19).

### Urinary biomarkers and association with AKI.

AKI occurred in 120 (13.9%) patients postoperatively. In general, preoperative levels were not associated with AKI, but patients with lower postoperative EGF and higher postoperative MCP-1 were more likely to develop AKI (Table 3). Higher postoperative urinary EGF levels were associated with decreased odds of AKI after full adjustment (adjusted OR [aOR] 0.55, 95% CI 0.44–0.70). Higher urinary MCP-1 levels were associated with increased odds of AKI after full adjustment (aOR 1.34, 95% CI 1.14–1.59). There was no interaction by AKI status on the association between postoperative EGF, MCP-1, or their ratio with the composite CKD outcome.

### EGF and CCL2 expression and regulation in kidney single-cell–based clusters of patients with AKI.

Cellular data from the 11 participants with AKI were obtained from the Kidney Precision Medicine Project (KPMP) kidney tissue atlas (Supplemental Table 6) (https://atlas.kpmp.org/repository). LDs were predominantly White, with a mean age of 45.1 ± 10.2 years, mean iothalamate GFR of 100.6 ± 16.9 mL/min/1.73 m^2^, and mean spot urine protein/creatinine ratio of 0.08 ± 0.04 g/g (20).

**Figure 3:**
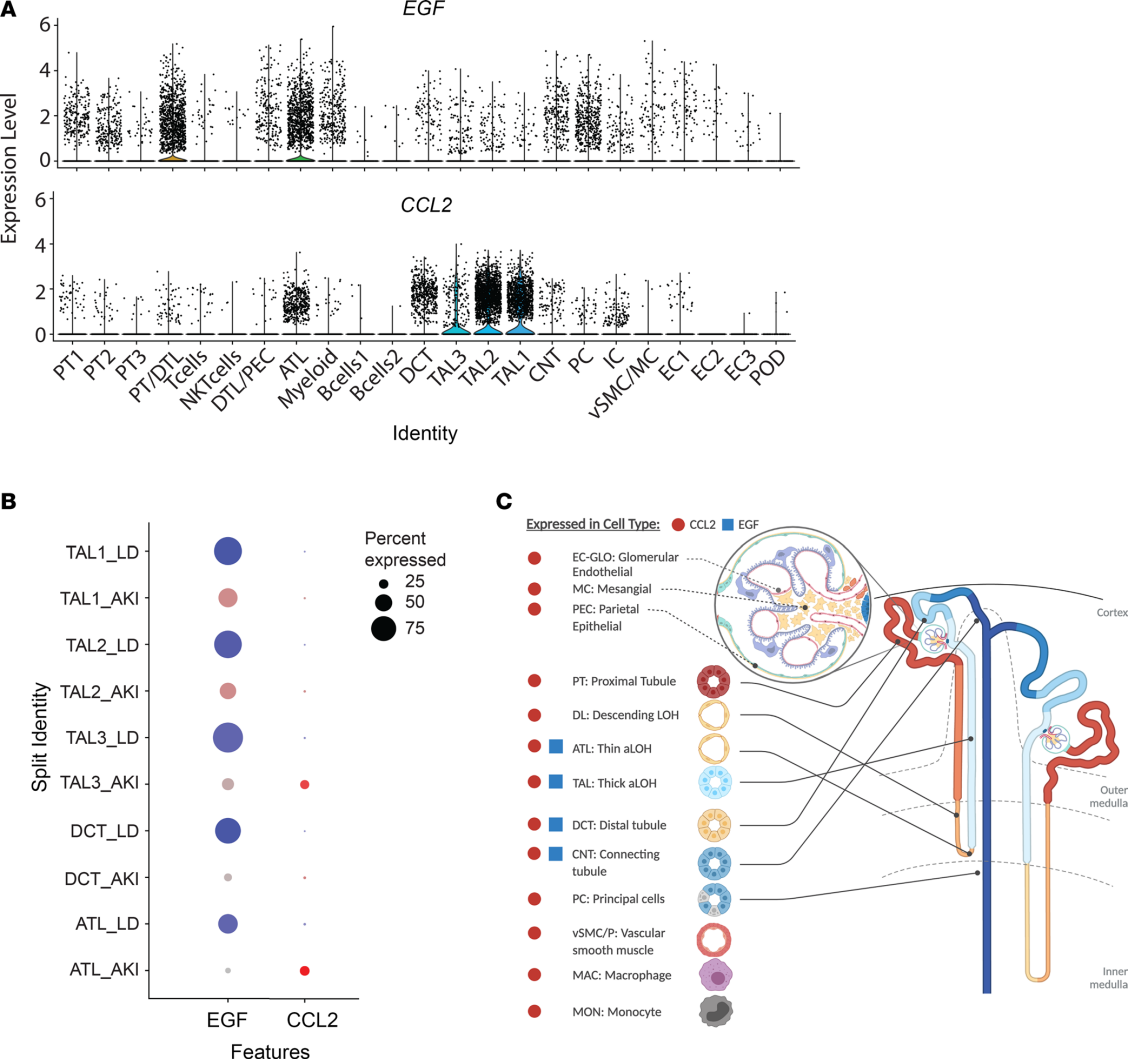
scRNA-Seq analysis of *EGF* and *CCL2* expression in kidney biopsy samples of patients with AKI. (**A**) Violin plots of *EGF* and *CCL2* expression in 23 cell clusters. (**B**) Dot plots showing the expression of *EGF* and *CCL2* in TAL, DCT, and ATL cell clusters from AKI (blue) and LD (red) kidney. Color intensity indicates expression level and the size of the dot indicates the percentage of cells expressing the gene. (**C**) Schematic illustration of a nephron, with segments that express *EGF* and *CCL2* in the scRNA-Seq data derived from patients with AKI indicated. ATL, ascending thin loop of Henle; *CCL2*, gene encoding MCP-1; CNT, connecting tubule; DCT, distal convoluted tubule; DTL, descending loop of Henle; EC, endothelial cell; IC, intercalated cell MC, mesangial cell; PC, principal cell; PEC, parietal epithelial cell; POD, podocyte; PT, proximal tubular epithelial cell; TAL, thick ascending loop of Henle; VSMC, vascular smooth muscle cell.

**Figure 4:**
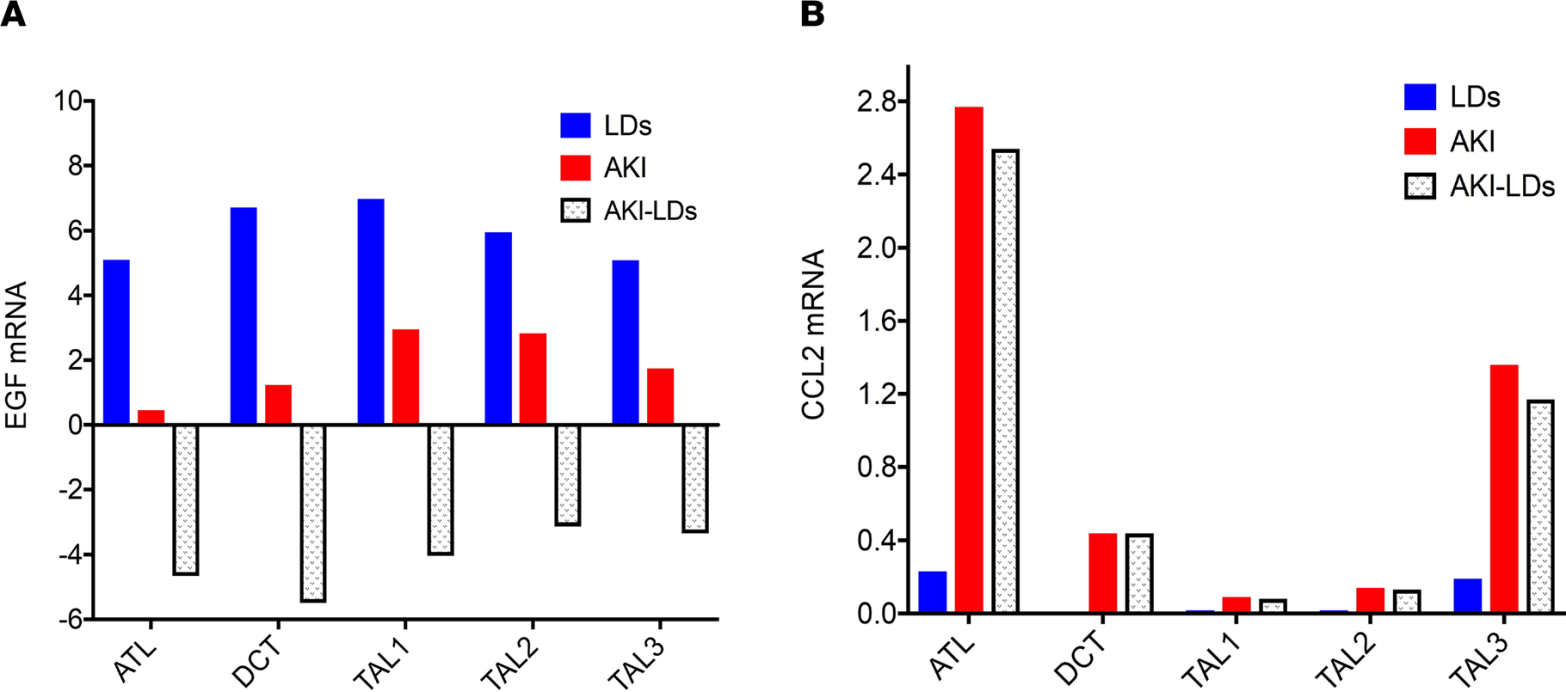
*EGF* and *CCL2* mRNA expression in different nephron cell clusters in AKI and LD cell populations. Average expression of *EGF* (**A**) and *CCL2* mRNA (**B**) and the changes in their expression (illustrated by stippled bars) between AKI (red) and LDs (blue) in the ATL, DCT, and TAL cell clusters.

A combined analysis of 57,995 cells from the 2 data sets defined 23 cell clusters (Figure 3) that covered the full spectrum of kidney cell types found along the nephron as well as tissue-resident immune cells. Restricted, cell type–enriched *EGF* mRNA expression was noted in the distal convoluted tubule (DCT), thick ascending loop of Henle (TAL), and thin ascending loop of Henle (ATL) cell clusters (Figure 3A), consistent with previous in situ hybridization studies (14). *EGF* was expressed in over 90% of TAL cells, 70% of DCT cells, and 50% ATL cells from LD tissues, whereas in protocol biopsies from patients with AKI, the percentage of *EGF*-expressing cells dropped below 50%, 10%, and 5% in the corresponding clusters (Figure 3B). Maximal reductions in both the percentage of cells expressing *EGF* and the average level of *EGF* expression were observed in DCT cells of patients with AKI compared with those of LDs. Figure 3C depicts *EGF* and *CCL2* expression in various kidney cell compartments. Cells aggregated into the DCT cell cluster were subsequently used to define the transcriptional characteristics of *EGF* expression (Figure 4).

*CCL2* was, as expected, highly expressed in myeloid cells in LD and AKI biopsies (21), but abundant *CCL2* expression was also observed in resident kidney cells in AKI, particularly in tubular compartments (Figure 3A). The greatest increase in both the percentage of cells expressing *CCL2* (Figure 3B) and the average level of *CCL2* (Figure 4) was observed in ATL cells, which were used to functionally characterize *CCL2*-expressing cells.

### Functional characterization of the EGF and CCL2 coregulated gene programs in tubular epithelial cells.

To define the transcriptional determinants of *EGF* and *CCL2* expression in kidney tubular cells, transcripts with significant differential regulation between DCT cells with *EGF* expression (*EGF*^+^) versus those without detectable *EGF* transcripts (*EGF*^–^), and between *CCL2*-expressing (*CCL2*^+^) cells versus nonexpressing (*CCL2*^–^) ATL cells, were mapped to their functional context. “Expressing” versus “nonexpressing” cells were based on greater than 0 (*CCL2* > 0 or *EGF* > 0) normalized gene expression. The bulk *EGF* and *CCL2* mRNA expression levels, as well as the ratio of *EGF/CCL2*, were significantly correlated with kidney function (eGFR) (Supplemental Figure 1).

### EGF coregulated gene programs in DCT cells.

We identified 234 genes as differentially expressed between *EGF*^–^ and *EGF*^+^ cells with an adjusted *P* value less than 0.05. Of the differentially expressed genes, 95% (223 genes) showed significantly lower expression in *EGF*^–^ cells. We clustered the *EGF*^–^ signature genes within the DCT functional network. The resulting modules (Supplemental Figure 2) contained key processes related to tubular function and the physiopathology of tubular injury, such as mitochondrial respiratory chain complex assembly (M1), metal ion homeostasis and cation homeostasis (M2), methylation (M4), and regulation of histone modification (M3). Importantly, the transmembrane receptor protein tyrosine kinase signaling pathway (M5) associated with cell survival and adhesion was enriched among the transcripts lost with *EGF* loss in *EGF*^–^ cells.

Fifty-one canonical pathways were significantly enriched in *EGF*^–^ signature genes in Ingenuity Pathway Analysis (IPA; QIAGEN). In addition to ErbB signaling, focal adhesion kinase signaling, and mitochondrial dysfunction pathways, which are consistent with the HumanBase functional network analysis, the pathway analysis also revealed that the insulin receptor and secretion signaling, integrin signaling, and sirtuin signaling pathways were among the top 20 most significantly enriched canonical pathways (Supplemental Table 7).

### CCL2 coregulated gene programs in ATL cells.

We identified 879 genes that were differentially expressed between *CCL2*^+^ cells and *CCL2*^–^ cells, with an adjusted *P* value less than 0.05. Ninety-eight percent (861 genes) showed substantially higher expression in *CCL2*^+^ cells. Transcripts coinduced with *CCL2* in *CCL2*^+^ cells at the level of the ATL mapped into gene modules enriched for key processes related to the physiopathology and underlying molecular mechanisms of tubular injury (Supplemental Figure 3). These processes include response to wounding (M3), response to hypoxia (M9), extracellular matrix accumulation–related (M4), kidney development–associated (M7), and cellular signal transduction pathways, including the NF-κB and JAK/STAT cascades (M5 and M8). Networks associated with translation processes were also enriched (M1 and M6).

Canonical pathways that were significantly enriched in *CCL2*^+^ gene signatures by IPA overlapped with the functional networks enriched in DCT with *EGF* loss (Supplemental Table 8). Activation of fibrotic pathways, actin cytoskeleton signaling, ERK/MAPK signaling, mammalian target of rapamycin (mTOR) signaling, and Rho family GTPase signaling were clearly associated with increased de novo expression of *CCL2*. Not surprisingly, the mitochondrial dysfunction and sirtuin signaling pathways were also among the top 20 most enriched canonical pathways.

The same analyses were also performed in the TAL cell cluster, a prominent site of altered gene expression during AKI (22). Nineteen out of the top 20 canonical pathways enriched in *CCL2*^+^ gene signatures in ATL were also significantly enriched in the TAL cell cluster. Nine out of the top 20 enriched canonical pathways in *EGF*^–^ signatures in the DCT were also significantly enriched in the TAL cluster. The shared canonical functions of *EGF*^–^ and *CCL2*^+^ signatures in TAL and ATL tubular cells are shown in Supplemental Tables 7 and 8, respectively.

### Upstream regulators of gene programs were associated with loss of EGF and increased CCL2 expression.

Next, we tested the differentially regulated gene sets for enrichment of common upstream regulators able to induce the differential transcriptional programs in *EGF*^–^ versus *EGF*^+^ cells of the DCT and *CCL2*^–^ versus *CCL2*^+^ cells in the ATL. Upstream regulators are molecules predicted to affect steady-state mRNA levels of dependent, downstream gene expression profiles. These upstream regulators include, but are not limited to, transcription factors, growth factors, kinases, and chemicals. Activities were predicted using literature-derived cause-and-effect relationships. In brief, upregulation of a transcription factor target gene supports a predicted activation of the transcription factor, whereas downregulation supports inhibition of the transcription factor (23, 24). For this study, we restricted the analysis to transcription factors and identified 6 common transcriptional regulators predicted to coregulate the *EGF* and *CCL2* gene programs. Based on activation *z* score, hypoxia-inducible factor 1 subunit α (HIF1A) was predicted to be a top upstream regulator mediating upregulation of transcripts in *CCL2*^+^ ATL cells, followed by forkhead box O1 (FOXO1), PPARG coactivator 1β (PPARGC1B), and SS18 subunit of BAF chromatin remodeling complex (SS18). These 3 upstream regulators were predicted to be inhibited as DCT cells lose *EGF* expression. Lysine demethylase 5A (KDM5A) showed the reverse pattern of activation compared with the aforementioned factors. The histone methyltransferase enhancer of zeste homolog-2 (EZH2) was the only transcription factor activated in both cell types (Supplemental Table 9).

## Discussion

In this prospective cohort study of adults undergoing cardiac surgery, we evaluated urinary EGF, MCP-1, and the EGF/MCP-1 ratio and found that higher postoperative levels of urinary EGF and EGF/MCP-1 were independently associated with a lower risk of the composite CKD outcome, whereas higher postoperative levels of urinary MCP-1 were associated with higher risk. We also observed that higher postoperative urinary EGF levels were independently associated with lower odds of AKI during hospitalization. We then used scRNA-Seq to define the tubular cell types that express *EGF* and *CCL2* and revealed, for the first time to our knowledge, the coregulated gene programs associated with loss of *EGF* and gain of *CCL2* expression in tubular cells under AKI conditions.

There are a number of mechanisms by which urinary EGF and MCP-1 may help distinguish patients at risk for both short-term AKI and long-term CKD outcomes. EGF is a 53–amnio acid polypeptide that serves as a growth factor implicated in repair after tissue injury (25). A number of retrospective studies have evaluated the role of urinary EGF as a predictive marker for kidney function decline in various clinical settings, such as diabetes mellitus, anti-neutrophil cytoplasmic antibody–associated vasculitis, and CKD in general (14, 26, 27). Specifically, lower levels of urinary EGF were associated with worse kidney prognosis, including eGFR decline and progression to end-stage kidney disease. In the setting of cardiac surgery, although postoperative EGF levels are generally lower than preoperative values, we speculate that a patient’s ability to maintain relatively normal postoperative EGF may indicate the patient’s ability to protect tubular function or to quickly recover differentiated function after kidney injury, indicating resilience against kidney injury. Moreover, the significant association of postoperative levels measured within 6 hours of surgery with AKI also makes urinary EGF a promising candidate marker of early kidney injury, even before a detectable rise in serum creatinine.

MCP-1 has been shown to mediate kidney injury through its role in macrophage recruitment (28), and elevated levels of both plasma and urinary MCP-1 have been shown to be important biomarkers of AKI (29, 30). We have previously shown that higher plasma MCP-1 levels were associated with increased AKI risk and death after cardiac surgery (30). Furthermore, higher urinary MCP-1 levels have been associated with chronic tubulointerstitial damage and CKD progression in various settings such as IgA nephropathy, diabetes mellitus, and obstructive uropathy (17, 19, 28, 31). Our current study provides additional evidence that higher levels of MCP-1 are related to increased expression of *CCL2* across various kidney cell types in the setting of kidney injury. Therefore, urinary MCP-1 has prognostic significance both for both short-term and long-term kidney function after cardiac surgery.

scRNA-Seq analysis of *EGF* and *CCL2* expression in kidney biopsies of healthy living donors and patients with AKI identified functional networks associated with loss of *EGF* and gain of *CCL2* in DCT and ATL cells. These 2 cell types exhibited dramatic changes in *EGF* and *CCL2* expression in LDs and AKI cells. *EGF*-associated transcriptional changes were linked to programs of ion homeostasis, histone modification, insulin receptor and secretion signaling, and integrin signaling. *CCL2*-associated transcriptional changes were linked to the cellular response to decreased oxygen levels, response to wound healing, JAK/STAT pathway dysregulation, and mTOR and Rho family GTPase signaling and dysfunction. Sirtuin signaling was linked to dysregulation of both the *EGF* and *CCL2* functional networks. These transcriptional networks underlie key processes that are involved in the physiopathology of tubular injury, including (but not limited to) cell survival, inflammation, and fibrosis. As urinary EGF and MCP-1 levels are significantly correlated with their corresponding *intrarenal* mRNA levels (14, 32), the identified networks also provide molecular mechanistic support for the association of urinary EGF and MCP-1 with the composite CKD outcome.

We observed an improved association with outcomes for the EGF/MCP-1 ratio in categorical analysis (aHR 0.50, 95% CI 0.33–0.74 for tertile 3 compared with tertile 1) compared with using either marker alone. Similar findings were reported in patients with diabetic kidney disease (17, 27, 33) and IgA nephropathy (19); however, a molecular interpretation for such improvement has not been provided. The transcriptional changes associated with the decreased expression of *EGF* and increased expression of *CCL2* provide a further rationale for a combination of these 2 urinary biomarkers, as they capture the key processes of tubular injury more comprehensively than either marker alone. As expected from the molecular analysis, the model using both markers yielded a more stable and reliable prediction of the composite CKD outcome.

The functional genomic analysis of the scRNA-Seq data is supported by analysis of bulk RNA isolated from the kidney tubulointerstitial compartments of patients with diabetic nephropathy, hypertensive nephropathy, or focal segmental glomerulosclerosis. The association between lower *EGF* and higher *CCL2* mRNA levels in the kidney tubulointerstitium suggests that the molecular pathways represented by these 2 biomarkers are simultaneously and actively involved in CKD pathogenesis.

Our candidate molecular pathway analysis identified the transcriptional regulators HIF1A, FOXO1, KDM5A, and EZH2, which could serve as future interventional targets. In response to stresses, including cardiac surgery in this study, these transcriptional regulators are either activated or inhibited in *CCL2*^+^ ATL cells and can lead to disruptive (mediated by EZH2) and compensative responses (by activation of HIF1A and FOXO1 and inhibition of KDM5A). Interestingly, in *EGF^–^* DCT cells, the disruptive responses (by EZH2 and KDM5A) are activated, but the compensative/adaptive factors HIF1A and FOXO1 are inhibited, suggesting that the transcriptional programs regulating prodisruptive responses dominated the compensative responses in *EGF*^–^ DCT cells, likely corresponding to an advanced stage of injury. Notably, the transcriptional modulator EZH2 was predicted to be activated upstream of both the *EGF*^–^ and *CCL2*^+^ dysregulated gene signatures upon AKI injury. EZH2 is known as a target for cancer therapy, and different types of EZH2 inhibitors have been developed (34). Recently, EZH2 has also been reported to play a crucial role in ischemia/reperfusion–induced AKI by regulating p38 signaling (35). Pharmacological inhibition of EZH2 protected against AKI through a mechanism associated with the preservation of adhesion/junctions and attenuation of the MAPK pathway (36). Our in silico models suggest that EZH2 may play an important role in loss of *EGF* in DCT cells and in increased de novo expression of *CCL2*, which is associated with the incidence of AKI and CKD, as well as progression of CKD. Thus, EZH2 may represent a potential target for interventions designed to mitigate or manage kidney injury at the time of surgery.

This study has a number of strengths and adds important information to the current state of knowledge regarding the established urinary biomarkers EGF and MCP-1. The associations of urinary EGF, MCP-1, and EGF/MCP-1 with the composite CKD outcome are in the same direction as observed in other clinical CKD settings (14). In this study, however, we demonstrated that these biomarkers measured around the time of cardiac surgery offer important prognostic information. Indeed, measurement of these biomarkers may improve risk stratification for both short-term AKI and long-term kidney outcomes in patients at the time of cardiac surgery. Furthermore, current AKI and CKD outcome predictors reflect 2 dimensions of kidney function, namely hemodynamics and glomerular filtration. However, kidney function is multidimensional and should be modeled as such. Adding parameters for tubular integrity (represented by urine EGF), inflammation and fibrosis (represented by MCP-1), and mitochondrial dysfunction and energy metabolism (represented by both), are critical next steps in the ongoing effort to obtain more precise and comprehensive insights on organ function and state. This study benefits from the use of robust clinical data, sample collection, processing, and retention beyond hospital discharge and follow-up. Furthermore, we performed a detailed adjustment of traditional risk biomarkers, including eGFR, urine albumin, and urine creatinine, which strengthens the robustness of our findings.

There are a number of limitations to this study. Most TRIBE-AKI Study participants were men and White, which limits the generalizability of our findings. Furthermore, we did not have information to ascertain the phenotype or etiology of CKD over the course of follow-up. The definition of incident CKD was based on a single postdischarge serum creatinine value. While many patients in the Canadian site had multiple postdischarge serum creatinine values, 30% patients at the U.S. study site did not have more than 1 follow-up serum creatinine after discharge. We do not have information on biomarker levels throughout follow-up to evaluate how these may have changed over time. The KPMP study participants who volunteered for the research biopsies used for the scRNA-Seq studies are being prospectively followed, but follow-up is not yet sufficient to ascertain progression to CKD in this cohort. We did not include urine output in the definition of AKI, which would likely have identified more AKI events.

In summary, we provide evidence for an association between postoperative urinary EGF, MCP-1, and EGF/MCP-1 with the composite CKD outcome and AKI after cardiac surgery. In the future, measurement of these urinary biomarkers may help risk stratification of patients following cardiac surgery and postdischarge follow-up for preventing and monitoring CKD events. The use of these biomarkers around the time of other known or predictable kidney insults may also help risk-stratify long-term CKD risk in other clinical settings. Our linkage of clinical associations with cell type–specific intrarenal pathways has opened up further avenues for mechanistic studies and the potential development of targeted therapies to prevent the AKI-to-CKD transition.

## Methods

### Study design, population, and data sources.

The TRIBE-AKI Study was a longitudinal prospective cohort study of adults who underwent cardiac surgery, either CABG or valvular surgery, and were deemed to be at high risk for AKI, across 6 academic centers in North America between 2007 and 2010. “High risk” for AKI was defined by the presence of 1 or more of the following: emergency surgery, preoperative serum creatinine > 2 mg/dL (>177 μmol/L), ejection fraction < 35% or grade 3 or 4 left ventricular dysfunction, age > 70 years, diabetes mellitus, concomitant CABG and valve surgery, or repeat revascularization surgery (18, 37). Detailed study enrollment methods have been described previously (37–39). Data from 2 recruitment sites, one in Ontario, Canada, and the other in New Haven, Connecticut, USA, were used for analysis in this substudy. Patients were included if they consented to linkage with administrative data for long-term follow-up and had at least 1 follow-up serum creatinine measurement after discharge.

### Sample collection and biomarker measurement.

Details of sample collection and processing were described previously (37). As part of the TRIBE-AKI protocol, urine and blood samples were collected preoperatively and then daily, for up to 5 days following cardiac surgery. Of note, immediate (first) postoperative urine and blood samples were collected within 6 hours of the end of the patient’s surgery and promptly centrifuged at 3000*g* for 10 minutes at 4°C. Urine supernatants were then aliquoted and stored in cryovials at –80°C until biomarker measurement, as described previously, using the Meso Scale Discovery (MSD) platform (Meso Scale Diagnostics), which combines electrochemiluminescence with multiarray technology (30, 37, 40, 41). For this study, we included preoperative and first postoperative urine samples for urinary EGF and MCP-1 measurements, measured simultaneously. EGF and MCP-1 were measured as part of multiplex assays using the MSD platform. Laboratory personnel were blinded to clinical data. Detection ranges for each biomarker were as follows: EGF (83.5 to 250,000 pg/mL); MCP-1 (2.1 to 12,500 pg/mL). Average interassay coefficients of variation were 9.4% and 13% for EGF and MCP-1, respectively.

### Kidney function evaluation and outcome definitions.

All preoperative serum creatinine values were measured within 2 months before surgery. Clinical AKI was defined as an increase at least 50% or 0.3 mg/dL in serum creatinine within the index hospitalization, using the preoperative serum creatinine as the baseline. Severity of AKI was classified by the Kidney Disease: Improving Global Outcomes staging criteria on the basis of the peak serum creatinine during the index hospitalization (42).

eGFRs were calculated using the Chronic Kidney Disease Epidemiology Collaboration Equation (43). The primary outcome of the study was a composite of CKD incidence or progression. In individuals with an eGFR at least 60 mL/min/1.73 m^2^ preoperatively, CKD incidence was defined as a 25% reduction in eGFR and a fall below 60 mL/min/1.73 m^2^. In individuals with an eGFR less than 60 mL/min/1.73 m^2^ preoperatively, CKD progression was defined as a 50% reduction in eGFR or a fall below 15 mL/min/1.73 m^2^. These definitions were based on established definitions, as outlined by the multicenter Assessment, Serial Evaluation, and Subsequent Sequelae in Acute Kidney Injury Study (ASSESS-AKI) (44, 45). Incidence or progression of CKD was determined after the index hospitalization, using serum creatinine measured after hospital discharge to determine eGFR.

For all patients enrolled at the Canadian study site, follow-up serum creatinine values were obtained using the Ontario Laboratories Information System, a province-wide integrated laboratory database incorporating outpatient and inpatient test results with serum creatinine values available 2007–2015. These data sets were linked using unique encoded identifiers and analyzed at ICES. Participants still alive on September 30, 2015, without the primary outcome were censored due to end of data availability.

For patients enrolled in the U.S. study site, follow-up creatinine measurements were ascertained through the Yale Joint Data Analytics Team’s Helix data repository, which includes data from all Yale New Haven Health–affiliated hospitals and outpatient practices with serum creatinine values available 2012–2018. Participants were censored at the time of their last serum creatinine measurement. In a subset of all participants (Canadian and American), home visits were conducted in the first year after discharge, and samples were collected for serum creatinine.

### Biopsy cohort for EGF and CCL2 scRNA-Seq analysis.

To define the RNA expression and regulation patterns of *EGF* and *CCL2* in kidney cells of patients with AKI, cell populations obtained by scRNA-Seq analysis of adult human kidney tissue samples from 11 patients with AKI and 18 LD adult human kidney tissue samples were clustered based on the transcription profiles of individual cells (Figure 3). AKI biopsies were obtained from hospitalized participants at 4 recruitment sites for the KPMP who consented to research biopsies: the Johns Hopkins Hospital, University of Pittsburgh Medical Center, Yale New Haven Hospital, and Columbia University Medical Center. LD cohort biopsies used as reference tissue were obtained before perfusion of LD kidneys and placement in the recipient.

Gene expression analysis was performed on renal biopsy specimens prospectively procured by European Renal cDNA Biobank (ERCB) for molecular analysis. ERCB is a European multicenter study capturing surplus renal biopsy tissue for gene expression analysis in RNA fixative (RNAlater, QIAGEN), coupled with cross-sectional clinical information collected at the time of a clinically indicated renal biopsy specimen (46). Biospecimens were collected after informed consent and with approval of the local ethics committee. The details of tissue harvesting, microdissection, RNA isolation, reverse transcription, linear amplification, target preparation, and data process followed published strategies (14, 47). Affymetrix GeneChip Human Genome U133A and U133 Plus 2.0 Array were used for this study. Gene expression data have been reported in previous studies (14, 48), and the Cel files were uploaded on Gene Expression Omnibus website (http://www.ncbi.nlm.nih.gov/geo/). Normalized tubulointerstitial compartment expression data of genes of interest were log_2_ -transformed and batch corrected. The correlation of log_2_-transformed transcript expression values with baseline eGFR (log_2_ transformed) was calculated using Pearson’s correlation.

Single-cell transcriptomes were generated with the biopsy core samples from cryopreserved AKI (11 biopsies) and LD (18 biopsies) samples. Tissue processing, single-cell isolation, and scRNA-Seq data generation were performed according to the protocol developed for the KPMP and are described in detail in the supplements of recent publications (49, 50) and online (51, 52).

### Identification of EGF and CCL2 coregulated gene signatures.

Genes that were differentially expressed in *EGF*-expressing (*EGF*^+^) versus nonexpressing (*EGF*^–^) DCT cells, and *CCL2*-expressing (*CCL2*^+^) versus nonexpressing (*CCL2*^–^) ATL cells, from patients with AKI were identified using the FindMarkers Seurat function. Expressing versus nonexpressing cells were based on greater than 0 (*CCL2* > 0 or *EGF* > 0) normalized gene expression. For the gene signature, all genes with Bonferroni-adjusted *P* values less than 0.05 were selected.

### Functional network analysis and candidate molecular pathway identification.

To determine the biological processes and pathways in the differentially expressed gene sets, we performed functional network clustering using Genome-scale Integrated Analysis of gene Networks 2.0 (53, 54). We projected gene signatures dysregulated in AKI into the HumanBase functional network representing biological processes and pathways being repressed in *EGF*^–^ DCT cells and *CCL2*^+^ ATL cells. We constructed the functional network by probabilistically integrating a large compendium of thousands of public omics data sets to predict the likelihood of 2 genes acting together in processes (53, 54). We performed community clustering in the network to identify tightly connected sets of genes using the HumanBase.io module detection function (55). We conducted upstream regulator analysis using QIAGEN’s IPA. Automated assessment of literature-curated cause-and-effect relationships were used to identify potential regulators of the shared transcriptional profile (24). This approach derived a prediction of activation or inhibition by identifying potential network changes that could explain the observed gene expression profile (activated) or reverse the observed expression profile (inhibited). To define candidate molecular pathways responsible for the differential regulation in cells with *EGF* loss and *CCL2* gain, we deployed an upstream regulator prediction approach and identified transcriptional regulators well-known for their roles in AKI or CKD, including several interventional targets such as HIF1A (56–59), FOXO1 (60–63), KDM5A (64), and EZH2 (34–36, 65).

### Statistics.

Descriptive characteristics were reported using mean (SD) or median (IQR) for continuous variables and frequency (percentage) for categorical variables. Cox proportional hazards regression was used to examine the association between both pre- and postoperative urinary biomarker levels and the composite CKD outcome, censoring for death before CKD incidence.

For unadjusted analyses, postoperative biomarker levels were modeled continuously after log_2_ transformation. In analyses using EGF/MCP-1, the absolute values of the biomarkers were used to calculate the ratio, with subsequent log_2_ transformation. Biomarkers were additionally modeled categorically in tertiles, with the lowest tertile serving as a reference group. Model 2 includes postoperative urine creatinine as a covariate, in order to adjust for biomarker concentration in the urine. Model 3 was further adjusted for age, sex, race, nonelective versus elective surgery, type of surgery, preoperative eGFR, diabetes, hypertension, congestive heart failure, myocardial infarction (MI), postoperative albuminuria, AKI stage, cardiopulmonary bypass time, and study site. Kolmogorov-type supremum tests were used to evaluate proportional hazards assumptions for all models. We also evaluated preoperative urinary EGF and MCP-1 with the composite outcome in separate analyses, adjusting for preoperative urinary creatinine and albuminuria.

We evaluated the association of postoperative urinary biomarkers with the development of AKI during the index hospitalization as a secondary outcome via logistic regression.

All analyses were performed in SAS (version 9.4; SAS Institute) and R (version 3.1.2; R Foundation for Statistical Computing). All tests of statistical significance were 2 tailed, with *P* < 0.05 considered significant. All scRNA-Seq data are accessible through the kidney atlas data biorepository website of the KPMP at https://atlas.kpmp.org/repository

### Study approval.

Institutional review boards at each participating site of the TRIBE-AKI Consortium and the KPMP approved the study, and written informed consent was obtained from all participants prior to inclusion in the study.

## Author contributions

SM and CRP led all stages of the work in collaboration with DGM, AXG, HTP, EM, YJ, WO, SGM, JLK, MGS, SGC, WJ, RM, and MK. AXG, SGC, and CRP were responsible for the study design of the TRIBE Cohort. HTP, EM, and YJ contributed significantly to data analysis and preparation of figures. WJ, RM, and MK performed the transcriptome analysis on AKI biopsies. SM, WJ, RM, DGM, HTP, EM, YJ, WO, SGM, JLK, MGS, SGC, AXG, ASB, JAK, MK, CRP contributed to drafting and critical revision of the manuscript for intellectual content before approving the final version. SM and WJ contributed equally to this project and serve as co–first authors. SM was responsible for the initial draft of the manuscript and all final edits along with CRP and so is listed first.

## Supplementary Material

Supplemental data

Trial reporting checklists

ICMJE disclosure forms

## Figures and Tables

**Table 1:**
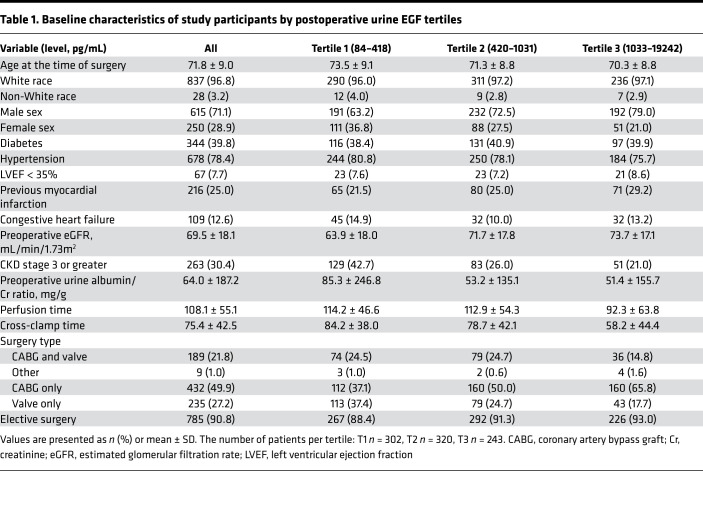
Baseline characteristics of study participants by postoperative urine EGF tertiles

**Table 2:**
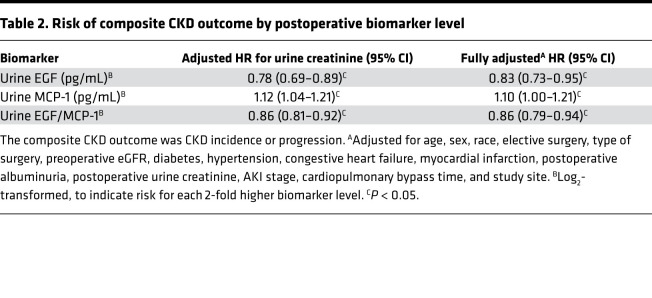
Risk of composite CKD outcome by postoperative biomarker level

**Table 3:**
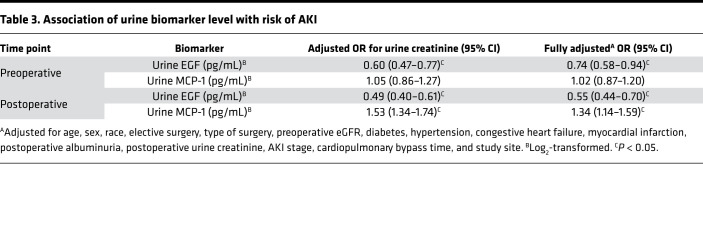
Association of urine biomarker level with risk of AKI

## Version 1 05/11/2021

In-Press Preview

## Version 2 06/08/2021

Electronic publication
