# Supplementary material for: Urinary EGF and MCP-1 and risk of CKD after cardiac surgery
Source: JCI Insight. 2021 Jun 8;6(11):e147464. doi: 10.1172/jci.insight.147464 (PMC8262289; doi:10.1172/jci.insight.147464)
Supplement: Supplemental data [file jciinsight-6-147464-s254.pdf]

SUPPLEMENTARY MATERIAL

Urinary EGF and MCP-1 and risk of CKD after cardiac surgery

Steven Menez, MD, MHS*[1]; Wenjun Ju, PhD*[2]; Rajasree Menon, PhD[2]; Dennis G. Moledina, MBBS, PhD[3,4]; Heather Thiessen-Philbrook, MMath[1]; Eric McArthur, MSc[5]; Yaqi Jia, MPH[1]; Wassim Obeid, PhD[1]; Sherry G. Mansour, DO, MS[3,4]; Jay L. Koyner, MD[6]; Michael G. Shlipak, MD, MPH[7]; Steven G. Coca, DO, MS[8]; Amit X. Garg, MD, PhD[5,9,10]; Andrew S. Bomback, MD[11]; John A. Kellum, MD[12]; Matthias Kretzler, MD[2]; Chirag R. Parikh, MD, PhD[1] for the TRIBE-AKI Consortium and the Kidney Precision Medicine Project

1. Division of Nephrology, Department of Medicine, Johns Hopkins University School of Medicine, Baltimore, Maryland

2. Division of Nephrology, Department of Medicine, Department of Computational Medicine and Bioinformatics, University of Michigan, Ann Arbor, Michigan

3. Section of Nephrology, Department of Internal Medicine, Yale University School of Medicine, New Haven, Connecticut

4. Clinical and Translational Research Accelerator, Department of Internal Medicine, Yale University School of Medicine, New Haven, Connecticut

5. ICES, Ontario, Canada

6. Section of Nephrology, University of Chicago, Chicago, Illinois

7. Kidney Health Research Collaborative and Division of General Internal Medicine, San Francisco Veterans Affairs Medical Center, University of California San Francisco, San Francisco, California

8. Division of Nephrology, Department of Medicine, Icahn School of Medicine at Mount Sinai, New York, New York

9. Division of Nephrology, Department of Medicine, Schulich School of Medicine and Dentistry, Western University, London, Ontario, Canada

10. Department of Epidemiology and Biostatistics, Schulich School of Medicine and Dentistry, Western University, London, Ontario, Canada

11. Division of Nephrology, Department of Medicine, Columbia University Vagelos College of Physicians and Surgeons, New York, NY

12. The Center for Critical Care Nephrology, Department of Critical Care Medicine, University of Pittsburgh, Pittsburgh, PA, USA

Supplemental Table 1. Baseline characteristics by post-operative urine MCP-1 tertiles				
**Variable (level, pg/ml)**	**All (N = 865)**	**Tertile 1 (4.7-143)**	**Tertile 2 (144-345)**	**Tertile 3 (346-11,683)**
Age at the time of surgery	71.8 ± 9.0	71.5 ± 9.7	71.8 ± 8.2	72.1 ± 9.1
White race	837 (96.8)	280 (94.6)	293 (98.0)	264 (97.8)
Male sex	615 (71.1)	194 (65.5)	221 (73.9)	200 (74.1)
Diabetes	344 (39.8)	112 (37.8)	142 (47.5)	90 (33.3)
Hypertension	678 (78.4)	228 (77.0)	229 (76.6)	221 (81.9)
LVEF <35%	67 (7.7)	18 (6.1)	18 (6.0)	31 (11.5)
Previous myocardial infarction	216 (25.0)	55 (18.6)	94 (31.4)	67 (24.8)
Congestive heart failure	109 (12.6)	34 (11.5)	31 (10.4)	44 (16.3)
Mean pre-operative eGFR, mL/min/1.73m^2	69.5 ± 18.1	71.3 ± 16.6	69.7 ± 18.3	67.4 ± 19.3
CKD stage 3 or greater	263 (30.4)	81 (27.3%)	92 (30.8)	90 (33.3)
Mean pre-operative urine albumin/Cr ratio, mg/g	64.0 ± 187.2	43.5 ± 105.1	80.6 ± 249.7	67.9 ± 174.8
Perfusion time	108.1 ± 55.1	97.1 ± 41.9	99.6 ± 49.4	130.3 ± 67.0
Cross-clamp time	75.4 ± 42.5	70.2 ± 34.5	68.3 ± 39.6	89.5 ± 50.1
Surgery type				
- CABG and valve	189 (21.8)	56 (18.9)	53 (17.7)	80 (29.6)
- Other	9 (1.0)	2 (0.7)	2 (0.7)	5 (1.9)
- CABG only	432 (49.9)	153 (51.7)	175 (58.5)	104 (38.5)
- Valve only	235 (27.2)	85 (28.7)	69 (23.1)	81 (30.0)
Elective surgery	785 (90.8)	276 (93.2)	268 (89.6)	241 (89.3)

Data are presented as mean ± SD and frequency (%) for continuous and categorical variables, respectively

CABG = coronary artery bypass graft; CKD = chronic kidney disease; Cr = creatinine; eGFR = estimated glomerular filtration rate; LVEF = left ventricular ejection fraction; MCP-1 = major chemoattractant protein 1.

The number of patients per tertile: T1 n=296, T2 n=299, T3 n=270

**Supplemental Table 2. CKD incidence or progression event rate**		
**Event**	**n/N**	**Event Rate (per 1000 person-years)**
Composite CKD outcome overall	266/865	55.4 (49.2-62.5)
- CKD incidence	212/602	64.7 (56.6-74.1)
- CKD progression	54/263	35.4 (27.1-46.3)
Composite CKD outcome in those with AKI	105/271	73.9 (61.0-89.4)
Composite CKD outcome in those without AKI	161/594	47.7 (40.8-55.6)

AKI = acute kidney injury; CKD = chronic kidney disease.

Supplemental Table 3. Association of post-operative urine biomarker tertiles with risk of composite CKD outcome			
**Biomarker**	**Tertile**	**Adjusted HR for Urine Creatinine (95% CI)**	**Fully Adjusted* HR (95% CI)**
Urine EGF (pg/mL)‡	Tertile 1 (83.5-417.8)	1.00 (referent)	1.00 (referent)
	Tertile 2 (420.5-1031)	0.84 (0.62-1.16)	0.91 (0.66-1.25)
	Tertile 3 (1033-19242)	0.59 (0.39-0.91) †	0.68 (0.43-1.07)
Urine MCP-1 (pg/mL)‡	Tertile 1 (4.7-143)	1.00 (referent)	1.00 (referent)
	Tertile 2 (144-345)	1.59 (1.15-1.22)†	1.46 (1.03-2.07)†
	Tertile 3 (346-11683)	1.68 (1.18-2.38)†	1.48 (0.99-2.22)
Urine EGF/MCP-1‡	Tertile 1 (0.01-1.77)	1.00 (referent)	1.00 (referent)
	Tertile 2 (1.78-5.42)	0.96 (0.72-1.26)	0.95 (0.70-1.29)
	Tertile 3 (5.44-108.73)	0.48 (0.34-0.67)†	0.50 (0.33-0.74)†

*Adjusted for age, sex, race, elective surgery, type of surgery, pre-operative eGFR, diabetes, hypertension, congestive heart failure, myocardial infarction, post-operative albuminuria, post-operative urine creatinine, AKI stage, cardiopulmonary bypass time, and study site
†: $P < 0.05$
‡: Log_2-transformed

**Supplemental Table 4. Risk of composite CKD outcome by pre-operative urine biomarker level**	
**Biomarker**	**Fully Adjusted* HR (95% CI)**
Urine EGF (pg/mL)‡	0.99 (0.85-1.15)
Urine MCP-1 (pg/mL)‡	1.23 (1.10-1.38)†
Urine EGF/MCP-1‡	0.79 (0.71-0.89)†

*Adjusted for age, sex, race, elective surgery, type of surgery, pre-operative eGFR, diabetes, hypertension, congestive heart failure, myocardial infarction, pre-operative urinary albumin, pre-operative urinary creatinine, and study site

†: $P < 0.05$

‡: Log_2-transformed

Supplemental Table 5. Risk of CKD incidence or progression by post-operative urine biomarker level, adjusted for pre-operative level	
**Biomarker**	**Fully Adjusted* HR (95% CI)**
Urine EGF (pg/mL)‡	0.84 (0.73-0.96)†
Urine MCP-1 (pg/mL)‡	1.08 (0.98-1.19)
Urine EGF/MCP-1‡	0.91 (0.83-0.99)†

* Adjusted for age, sex, race, elective surgery, type of surgery, pre-operative eGFR, diabetes, hypertension, congestive heart failure, myocardial infarction, post-operative albuminuria, post-operative urine creatinine, AKI stage, cardiopulmonary bypass time, and study site, and pre-operative biomarker level

†: $P < 0.05$

‡: Log_2-transformed

Supplemental Table 6. Basic clinical data of AKI patients included in the ScRNAseq analysis. Data derived from open access at KPMP.org							
**Tissue Type**	**Sex**	**Age (Years)**	**Race**	**KDIGO Stage**	**Baseline eGFR (ml/min/1.73m2)**	**Diabetes**	**Hypertension**
AKI	Male	30-39	Black or African-American	Stage 2	90-99	Yes	Yes
AKI	Female	70-79	Black or African-American	Stage 2	70-79	Yes	Yes
AKI	Female	30-39	White	Stage 3	110-119	No	No
AKI	Male	40-49	Black or African-American	Stage 3	70-79	Yes	Yes
AKI	Male	50-59	Black or African-American	Stage 3	100-109	No	No
AKI	Male	30-39	Don't Know	Stage 2	120-129	No	No
AKI	Male	60-69	White	Stage 2	60-69	Yes	No
AKI	Male	70-79	White	Stage 3	70-79	Don't Know	No
AKI	Male	60-69	White	Stage 3	80-89	Yes	Yes
AKI	Male	60-69	White	Stage 3	70-79	No	Yes
AKI	Male	20-29	White	Stage 3	70-79	No	No
AKI	Female	60-69	White	Stage 3	50-59	Yes	No
AKI	Female	50-59	White	Stage 1	70-79	No	No
AKI	Male	20-29	Black or African-American	Stage 2	70-79	No	No

Supplemental Table 7. Top 20 canonical pathways (by P value enrichment) enriched in EGF co-regulated transcripts in DCT cells of patients with AKI. Pathways are only included when more than 3 molecules in the particular pathway are present in the signature. * denotes pathways that are also significantly enriched in the same analysis in TAL cells			
**Ingenuity Canonical Pathways**	**Molecules**	**P value**	**Z-score**
Oxidative Phosphorylation*	MT-CO3,MT-ND1,MT-ND3,NDUFA13,NDUFA3,NDUFA7,NDUFB1	<0.001	-2.65
Mitochondrial Dysfunction*	BACE2,MT-CO3,MT-ND1,MT-ND3,NDUFA13,NDUFA3,NDUFA7,NDUFB1	<0.001	
ErbB Signaling	EGF,ERBB2,ERBB4,MAP2K6,PAK4,PIK3C2G	<0.001	-2.24
TR/RXR Activation*	ACACA,DIO2,NCOA4,PIK3C2G,THRA	0.001	
Sirtuin Signaling Pathway*	BCL2L11,GOT2,MT-ND1,MT-ND3,NDUFA13,NDUFA3,NDUFA7,NDUFB1,SCNN1A	0.002	2.83
PPARα/RXRα Activation*	ADIPOR1,GOT2,INSR,LPL,MAP2K6,PLCL1,STAT5B	0.002	-0.82
Telomerase Signaling	EGF,PIK3C2G,PPM1L,TERF2IP,TPP1	0.004	
Integrin Signaling	BCAR3,CAPN10,PAK4,PFN2,PIK3C2G,RHOQ,TSPAN7	0.004	-2.45
IL-15 Production	DSTYK,ERBB2,ERBB4,INSR,MAP2K6	0.006	-2.24
Insulin Secretion Signaling Pathway	GCGR,INSR,PIK3C2G,PLCL1,SCNN1A,STAT5B,VAMP2	0.009	-2.65
Semaphorin Neuronal Repulsive Signaling Pathway	ERBB2,MAP2K6,PAK4,PIK3C2G,SEMA6D	0.011	-0.45
Insulin Receptor Signaling	INSR,PIK3C2G,RHOQ,SCNN1A,VAMP2	0.011	-2.24
Senescence Pathway	CAPN10,MAP2K6,NF1,PHF1,PIK3C2G,PPM1L,RBL2	0.016	-1.13
FAK Signaling*	CAPN10,EGF,PAK4,PIK3C2G	0.017	
Neuregulin Signaling	EGF,ERBB2,ERBB4,STAT5B	0.018	-2.00
Sperm Motility	DSTYK,ERBB2,ERBB4,INSR,MAP2K6,PLCL1	0.020	
Pancreatic Adenocarcinoma Signaling	EGF,ERBB2,PIK3C2G,VEGFA	0.020	-2.00
Glioblastoma Multiforme Signaling	EGF,NF1,PIK3C2G,PLCL1,RHOQ	0.021	-2.00
Actin Cytoskeleton Signaling*	CYFIP2,EGF,KNG1,PAK4,PFN2,PIK3C2G	0.022	
LXR/RXR Activation	ACACA,HADH,KNG1,LPL	0.028	
Growth Hormone Signaling	IGFBP3,PIK3C2G,STAT5B	0.030	
Xenobiotic Metabolism CAR Signaling Pathway	ALDH5A1,ALDH6A1,HS6ST2,MAP2K6,PPM1L	0.035	-1.34
ILK Signaling	MAP2K6,PIK3C2G,PPM1L,RHOQ,VEGFA	0.035	-1.34
STAT3 Pathway	EGF,INSR,NDUFA13,VEGFA	0.040	-1.00
Axonal Guidance Signaling	EGF,ERBB2,NTN4,PAK4,PFN2,PIK3C2G,PLCL1,SEMA6D,VEGFA	0.047	

Supplemental Table 8. Top 20 canonical pathways (by *P* value enrichment) enriched in *CCL2* co-regulated transcripts in ATL cells of patients with AKI. Pathways are only included when more than 3 molecules in the particular pathway are present in the signature. * denotes pathways that are also significantly enriched in the same analysis in TAL cells			
**Ingenuity Canonical Pathways**	**Molecules**	**P value**	**Z-score**
Hepatic Fibrosis Signaling Pathway	ATF4,CALM1 (includes others),CCL2,CCN2,CD40,CEBPB,CXCL8,EDN1,FGFR1,GNAI3,HIF1A,ICAM1,IKBKB,ITGA3,ITGAV,ITGB1,ITGB3,MYC,MYL12A,MYL12B,MYL6,MYL9,MYLK,NFKB2,NFKBIA,PDGFB,PRKCA,PRKCI,RAC1,RAP1B,RASD1,RHOB,RHOC,RHOG,RND3,RRAS2,SOD2,SPP1,STAT3,TGFB1	3.4E-09	5.284
EIF2 Signaling*	ACTB,ATF4,ATF5,EIF1,EIF2S2,EIF3A,EIF3G,EIF3J,EIF3M,EIF4A1,EIF4G1,EIF4G2,FAU,MYC,PABPC1,PPP1CA,PPP1CB,RAP1B,RASD1,RPL11,RPL12,RPL14,RPL18A,RPL19,RPL22,RPL22L1,RPL23,RPL24,RPL26,RPL27,RPL28,RPL29,RPL31,RPL32,RPL34,RPL35,RPL35A,RPL36,RPL36AL,RPL37,RPL38,RPL39,RPL41,RPL7,RPL7A,RPL8,RPLP0,RPLP1,RPLP2,RPS12,RPS13,RPS15,RPS15A,RPS16,RPS17,RPS18,RPS19,RPS21,RPS23,RPS24,RPS25,RPS26,RPS27A,RPS27L,RPS28,RPS7,RRAS2	1.3E-41	5.096
Integrin Signaling*	ACTB,ACTG1,ACTN1,ACTN4,ACTR3,ARF3,ARF4,ARF5,ARPC1B,ARPC2,ARPC3,ARPC4,ARPC5,ARPC5L,BCAR1,CAPNS1,CAV1,ILK,ITGA3,ITGAV,ITGB1,ITGB3,ITGB6,MYL12A,MYL12B,MYL9,MYLK,NEDD9,PDGFB,PFN1,PPP1CB,RAC1,RAP1B,RASD1,RHOB,RHOC,RHOG,RND3,RRAS2,VCL,ZYX	4.0E-18	5.096
Actin Cytoskeleton Signaling*	ACTB,ACTG1,ACTN1,ACTN4,ACTR3,ARPC1B,ARPC2,ARPC3,ARPC4,ARPC5,ARPC5L,BCAR1,BRK1,CFL1,DIAPH1,DIAPH3,FLNA,ITGA3,ITGAV,ITGB1,ITGB3,MSN,MYH9,MYL12A,MYL12B,MYL6,MYL9,MYLK,PDGFB,PFN1,PPP1CB,RAC1,RAP1B,RASD1,RRAS2,SLC9A1,TMSB10/TMSB4X,TRIO,VCL	1.6E-15	5.048
Signaling by Rho Family GTPases*	ACTB,ACTG1,ACTR3,ARPC1B,ARPC2,ARPC3,ARPC4,ARPC5,ARPC5L,CDC42EP1,CFL1,DIAPH3,GNAI3,GNB2,ITGA3,ITGAV,ITGB1,ITGB3,MSN,MYL12A,MYL12B,MYL6,MYL9,MYLK,NFKB2,PARD3,PRKCI,RAC1,RHOB,RHOC,RHOG,RND3,SLC9A1,STMN1,VIM	2.5E-11	4.536
Ephrin Receptor Signaling*	ACTR3,ARPC1B,ARPC2,ARPC3,ARPC4,ARPC5,ARPC5L,ATF4,BCAR1,CFL1,EFNA1,EFNB2,EPHA2,EPHB2,GNAI3,GNB2,ITGA3,ITGAV,ITGB1,ITGB3,PDGFB,PTPN11,RAC1,RAP1B,RASD1,RGS3,RRAS2,SDCBP,STAT3	1.1E-10	4.146
ILK Signaling*	ACTB,ACTG1,ACTN1,ACTN4,ATF4,CFL1,FLNA,HIF1A,ILK,IRS1,ITGB1,ITGB3,ITGB6,KRT18,MYC,MYH9,MYL6,MYL9,NFKB2,PPP1R14B,PPP2R5A,RAC1,RHOB,RHOC,RHOG,RND3,TMSB10/TMSB4X,VCL,VIM	1.3E-10	4.041
Actin Nucleation by ARP-WASP Complex*	ACTR3,ARPC1B,ARPC2,ARPC3,ARPC4,ARPC5,ARPC5L,ITGA3,ITGAV,ITGB1,ITGB3,RAC1,RAP1B,RASD1,RHOB,RHOC,RHOG,RND3,RRAS2	1.1E-10	4

Rac Signaling*	ACTR3,ARPC1B,ARPC2,ARPC3,ARPC4,ARPC5,ARPC5L,BRK1,CD44,CFL1,ITGA3,ITGAV,ITGB1,ITGB3,NFKB2,PARD3,PRKCI,RAC1,RAP1B,RASD1,RRAS2	4.4E-09	4
Regulation of Actin-based Motility by Rho*	ACTB,ACTR3,ARPC1B,ARPC2,ARPC3,ARPC4,ARPC5,ARPC5L,CFL1,ITGA3,ITGAV,ITGB1,ITGB3,MYL12A,MYL12B,MYL6,MYL9,MYLK,PFN1,PPP1CB,RAC1,RHOB,RHOC,RHOG,RND3	5.0E-14	3.838
mTOR Signaling*	EIF3A,EIF3G,EIF3J,EIF3M,EIF4A1,EIF4B,EIF4EBP1,EIF4G1,EIF4G2,FAU,FKBP1A,HIF1A,IRS1,PPP2R5A,PRKCA,PRKCI,RAC1,RAP1B,RASD1,RHEB,RHOB,RHOC,RHOG,RND3,RPS12,RPS13,RPS15,RPS15A,RPS16,RPS17,RPS18,RPS19,RPS21,RPS23,RPS24,RPS25,RPS26,RPS27A,RPS27L,RPS28,RPS7,RRAS2	4.0E-19	3.5
RhoA Signaling*	ACTB,ACTG1,ACTR3,ARPC1B,ARPC2,ARPC3,ARPC4,ARPC5,ARPC5L,CDC42EP1,CFL1,MSN,MYL12A,MYL12B,MYL6,MYL9,MYLK,PFN1,PPP1CB,RHPN2,RND3	5.9E-09	3.273
Remodeling of Epithelial Adherens Junctions*	ACTB,ACTG1,ACTN1,ACTN4,ACTR3,ARPC1B,ARPC2,ARPC3,ARPC4,ARPC5,ARPC5L,HGS,NME1,TUBA1A,TUBB,VCL,ZYX	3.6E-10	2.828
ERK/MAPK Signaling*	ATF4,BCAR1,DUSP1,EIF4EBP1,ELF3,ETS1,H3-3A/H3-3B,HSPB1,ITGA3,ITGAV,ITGB1,ITGB3,MYC,PPP1CA,PPP1CB,PPP1R14B,PPP2R5A,PRKCA,PRKCI,RAC1,RAP1B,RASD1,RRAS2,STAT1,STAT3,YWHAB,YWHAG,YWHAZ	2.5E-09	2.132
Regulation of eIF4 and p70S6K Signaling*	EIF1,EIF2S2,EIF3A,EIF3G,EIF3J,EIF3M,EIF4A1,EIF4EBP1,EIF4G1,EIF4G2,FAU,IRS1,ITGA3,ITGAV,ITGB1,ITGB3,PABPC1,PPP2R5A,RAP1B,RASD1,RPS12,RPS13,RPS15,RPS15A,RPS16,RPS17,RPS18,RPS19,RPS21,RPS23,RPS24,RPS25,RPS26,RPS27A,RPS27L,RPS28,RPS7,RRAS2	1.3E-19	1.633
Sirtuin Signaling Pathway*	ACLY,APEX1,BAX,CXCL8,EPAS1,GABARAP,GLS,H3-3A/H3-3B,HIF1A,MAP1LC3B,MYC,NAMPT,NDUFA2,NDUFA6,NDUFB3,NDUFB4,NDUFB6,NDUFS5,NDUFS6,NDUFS7,NDUFS8,NFKB2,PARP1,PGK1,POLR1D,POLR2F,SLC25A5,SLC2A1,SMARCA5,SOD2,STAT3,TIMM10,TIMM13,TIMM17A,TIMM8B,TUBA1A,VDAC1	1.0E-10	-1.4
Coronavirus Pathogenesis Pathway*	ATF4,BAX,CCL2,CXCL8,EEF1A1,FAU,IKBKB,KPNB1,NFKB2,NFKBIA,NPM1,PA2G4,RPS12,RPS13,RPS15,RPS15A,RPS16,RPS17,RPS18,RPS19,RPS21,RPS23,RPS24,RPS25,RPS26,RPS27A,RPS27L,RPS28,RPS7,STAT1,STAT3,TGFB1	1.0E-15	-3.053
RhoGDI Signaling*	ACTB,ACTG1,ACTR3,ARPC1B,ARPC2,ARPC3,ARPC4,ARPC5,ARPC5L,CD44,CFL1,GNAI3,GNB2,ITGA3,ITGAV,ITGB1,ITGB3,MSN,MYL12A,MYL12B,MYL6,MYL9,PRKCA,RAC1,RHOB,RHOC,RHOG,RND3	5.4E-10	-4.491
Epithelial Adherens Junction Signaling*	ACTB,ACTG1,ACTN1,ACTN4,ACTR3,ARPC1B,ARPC2,ARPC3,ARPC4,ARPC5,ARPC5L,FGFR1,MYH9,MYL6,MYL9,PARD3,RAC1,RAP1B,RASD1,RRAS2,TUBA1A,TUBB,VCL,ZYX	2.5E-09	
Mitochondrial Dysfunction*	COX17,COX6B1,COX7B,COX7C,CYCS,NDUFA2,NDUFA6,NDUFB3,NDUFB4,NDUFB6,NDUFS5,NDUFS6,NDUFS7,NDUFS8,OGDH,PARK7,PRDX3,PRDX5,PSENEN,SOD2,UQCR10,UQCRB,UQCRH,UQCRQ,VDAC1	5.9E-09	

**Supplemental Table 9. Common upstream regulators of *EGF* and *CCL2* coregulated transcripts in DCT and ATL cells**				
**Upstream Transcriptional Regulators**	**Down-regulated Gene Signature in EGF$^-$ DCT Cells**		**Up-regulated Gene Signature in CCL2$^+$ ATL Cells**	
	**Activation Z-score**	***P*-value of Overlap**	**Activation *Z*-score**	***P*-value of Overlap**
HIF1A	-1.89	0.0114	4.793	<0.0001
FOXO1	-2.18	0.0060	3.286	<0.0001
PPARGC1B	-2.21	0.0001	2.607	0.0017
SS18	-2.00	0.0004	2.236	0.0116
KDM5A	2.24	0.0090	-3.499	<0.0001
EZH2	2.20	0.0163	1.944	<0.0001

ATL = ascending thin loop of Henle; *CCL2* = gene encoding monocyte cheomoattractant protein-1 (MCP-1); DCT = distal convoluted tubule; EZH2 = Enhancer of zeste 2 polycomb repressive complex 2 subunit; FOXO1 = Forkhead box O1; HIF1A = Hypoxia-inducible factor 1 subunit alpha; KDM5A = Lysine Demethylase 5A; PPARGC1B = Peroxisome Proliferator Activated Receptor Gamma Coactivator 1 Beta; SS18 = SS18 Subunit Of BAF Chromatin Remodeling Complex.

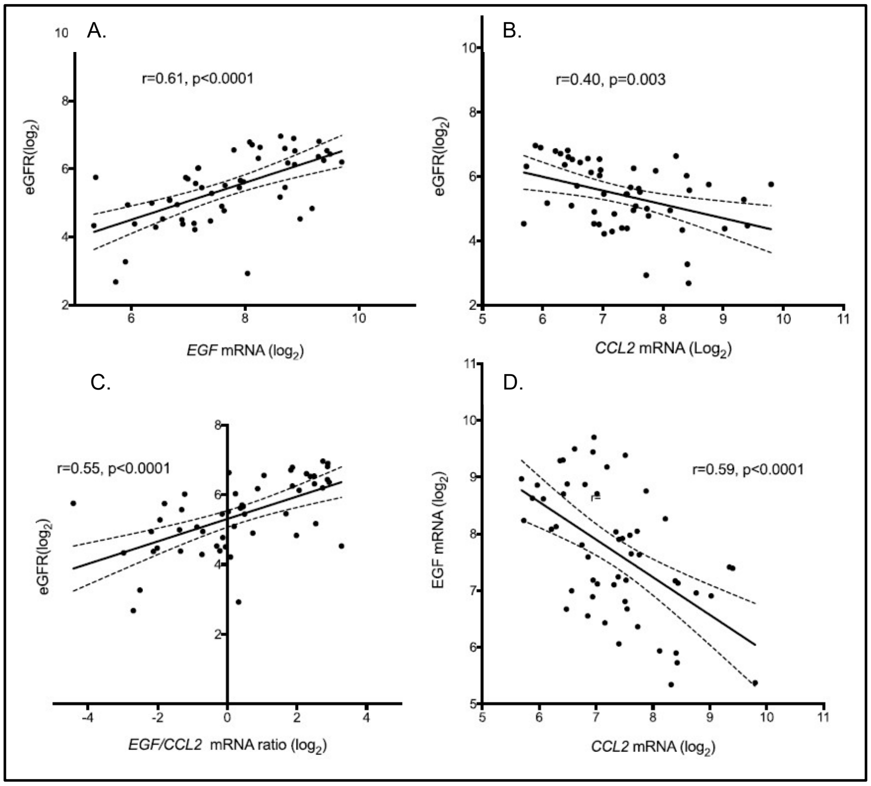

Supplemental Figure 1. Association of *EGF* and *CCL2* mRNA levels in kidney tissue of patients with chronic kidney disease (diabetic kidney disease, hypertension-attributed kidney disease, and focal segmental glomerulosclerosis), and their correlation with eGFR. (A) *CCL2* = gene encoding monocyte chemoattractant protein-1 (MCP-1); *EGF* = epidermal growth factor; eGFR = estimated glomerular filtration rate.

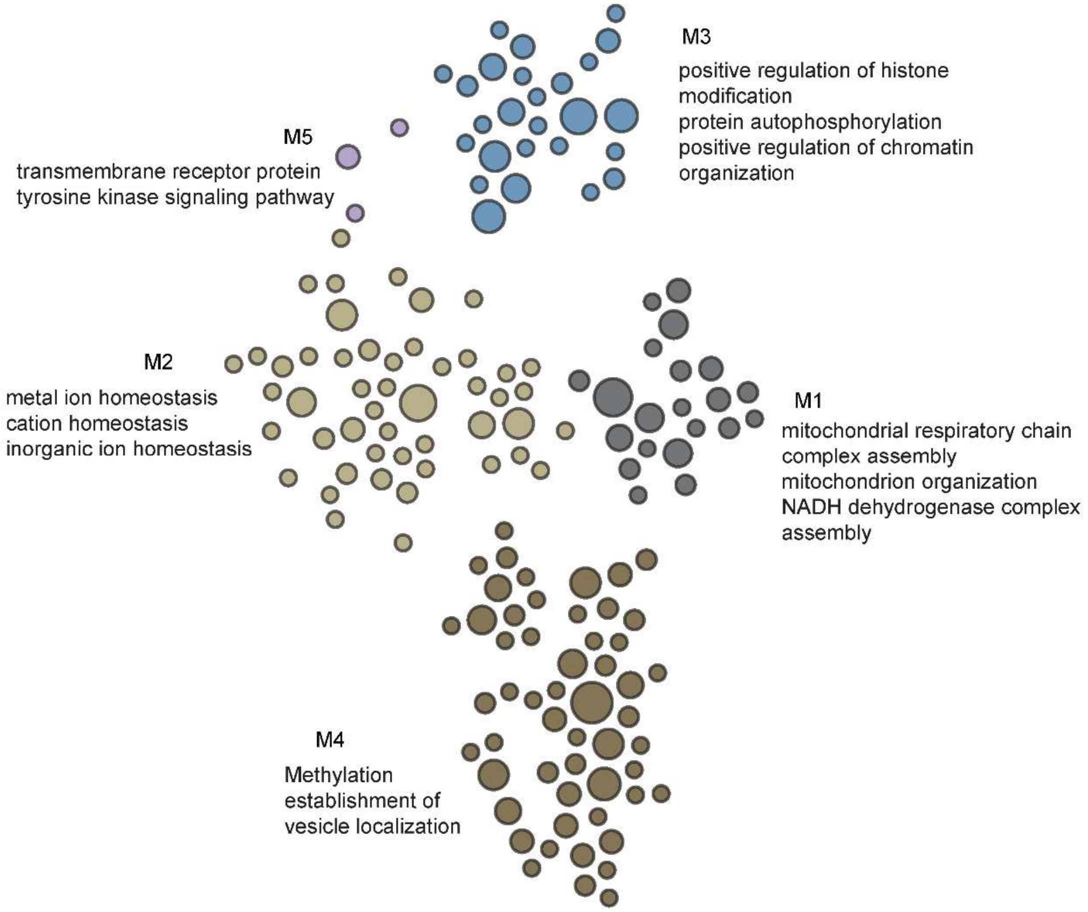

Supplemental Figure 2. Functional summaries of repressed signatures in *EGF*[-] DCT cells in patients with AKI. Functional summary of repressed signature in *EGF*[-] DCT cells of patients with AKI – community clustering and enriched processes of the genes that are significantly down regulated in DCT cells that do not express *EGF* compared to *EGF*-expressing DCT cells. Each dot represents input gene, and the size of the dot indicates relative connectivity.

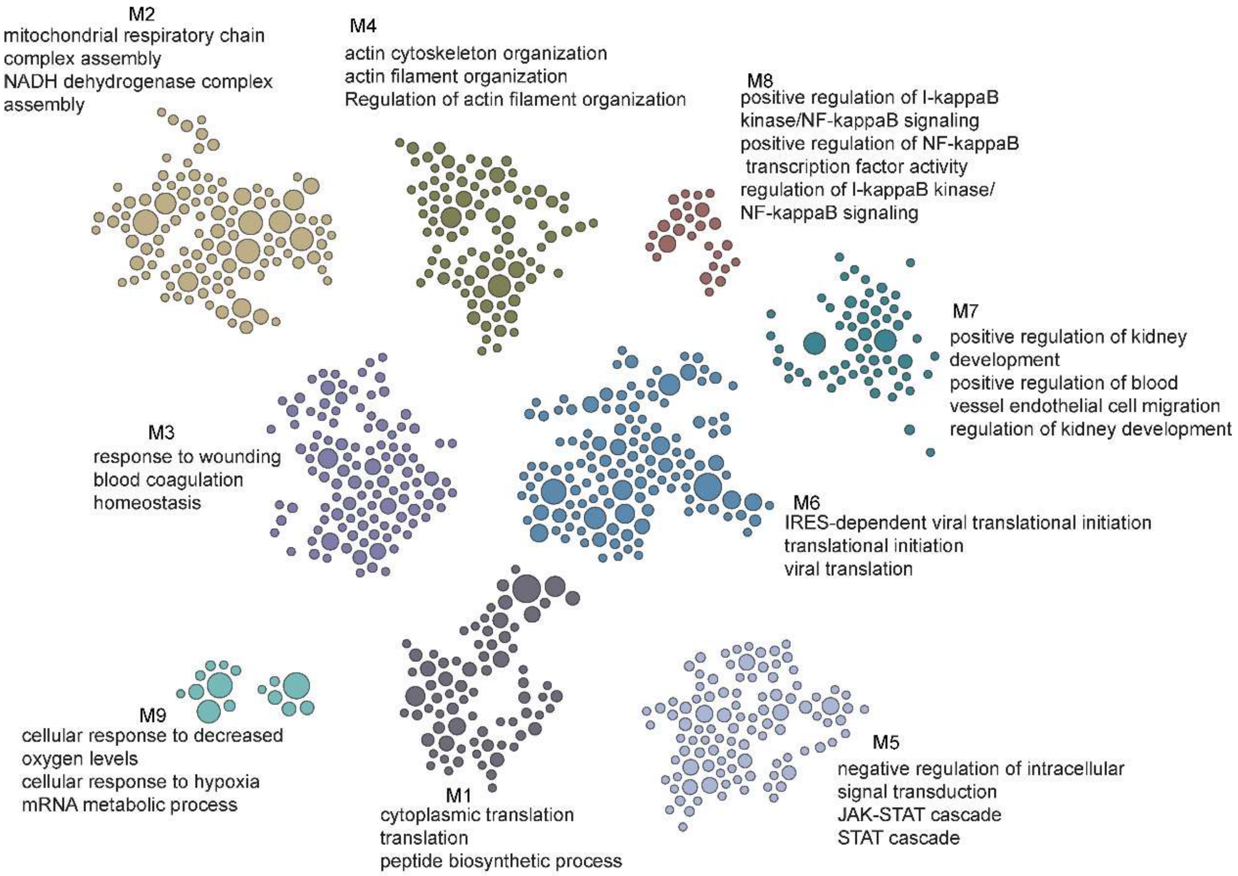

Supplemental Figure 3. Functional summaries of repressed signatures in *CCL2*⁺ ATL cells in patients with AKI. Functional summary of *CCL2*+ expression signature – community clustering and enriched processes of the genes that are significantly up-regulated in *CCL2*-expressing+ ATL cells compared to those that do not. NADH = nicotinamide adenine dinucleotide hydride; JAK-STAT= Janus kinase (JAK) - signal transducer and activator of transcription protein (STAT). Each dot represents input gene, and the size of the dot indicates relative connectivity.

Supplemental Acknowledgements

For the Kidney Precision Medicine Project

American Association of Kidney Patients, Tampa, FL: Richard Knight
Beth Israel Deaconess, Boston, MA: Stewart Lecker, Isaac Stillman
Boston Cell Standards, Boston, MA: Steve Bogen
Boston Medical Center, Boston, MA: Afolarin A. Amodu, Titlayo Ilori, Shana Maikhor, Insa Shmidt
Boston University, Boston, MA: Sushrut Waikar, Laurence H. Beck, Joel M . Henderson, Ingrid Onul, Ashish Verma
Brigham & Women's Hospital, Boston, MA: Gearoid McMahon, Astrid Weins, Samuel Short, Mia R. Colona, M. Todd Valerius
Broad Institute, Cambridge, MA: Anna Greka, Nir Hacohen, Paul Hoover, Jamie L. Marshall
Case Western Reserve, Cleveland, OH: Mark Aulisio, Yijiang M. Chen, Andrew Janowczyk, Catherine Jayapandian, Vidya S. Viswanathan, William S. Bush, Dana C. Crawford, Anant Madabhushi
Cleveland Clinic, Cleveland, OH: Lakeshia Bush, Leslie Cooperman, Agustin Gonzalez-Vicente, Leal Herlitz, John O'toole, Emilio Poggio, John Sedor, Stacey Jolly, Ellen Palmer, Dianna Sendrey, Kassandra Spates-Harden, Jonathan Taliercio
University of Colorado, Boulder, CO: Petter M. Bjornstad, Laura Pyle, Carissa Vinovskis
Columbia University, New York, NY: Paul Appelbaum, Olivia Balderes, Jonathan Barasch, Andrew Bomback, Pietro A. Canetta, Vivette D. D'Agati, Krzysztof Kiryluk, Satoru Kudose, Karla Mehl, Jai Radhakrishnan, Chenhua Weng, Ning Shang
Duke University, Durham, NC: Shweta Bansal
European Molecular Biology Laboratory, Heidelberg, Germany: Theodore Alexandrov
Harvard University, Boston, MA: Helmut Rennke
Indiana University, Indianapolis, IN: Tarek M. El-Achkar, Daria Barwinska, Sharon Bledsoe, Katy Borner, Andreas Bueckle, Yinghua Cheng, Pierre Dagher, Kenneth Dunn, Michael Eadon, Michael Ferkowicz, bruce W. Herr, Katherine Kelly, Ricardo Melo Ferreira, Ellen M. Quardokus, Elizabeth Record, Marcelino Rivera, Jing Su, Timothy Sutton, Seth Winfree, James C. Williams,, Jr
John Hopkins University, Baltimore, MD: Steven Menez, Chirag Parikh, Avi Rosenberg, Pam Villalobos, Rubab Malik, Derek Fine, Mohammed Atta, Jose Manuel Monroy Trujillo, Yumeng Weng
Joslin Diabetes Center, Boston, MA: Camille Johansen, Alison Slack, Sylvia Rosas, Mark Williams, Neil Roy, Jennifer Sun
Mount Sinai, New York, NY: Evren Azeloglu, Cijang (John) He, Ravi Iyengar, Jens Hansen, Yuguang Xiong
Northshore: Pottumarthi Prasad
Northwestern University, Chicago, IL: Anand Srivastava
Ohio State University, Columbus, OH: Samir Parikh, Brad Rovin, Sethu M. Madhavan, John P. Shapiro
Pacific Northwest National Laboratories, Richland, WA: Chris Anderton, Ljiljana Pasa-Tolic, Dusan Velickovic, Jessica Lukowski
Parkland Center for Clinical Innovation, Dallas, TX: George (Holt) Oliver
Patient Partners: Joseph Ardayfio, Jack Bebiak, Keith Brown, Taneisha Campbell, Catherine Campbell, Lynda Hayashi, Nichole Jefferson, Robert Koewler, Glenda Roberts, John Saul, Anna Shpigel, Edith Christine Stutzke, Lorenda Wright, Leslie Miegs, Roy Pinkeney
Princeton University, Princeton, NJ: Rachel Sealfon, Olga Troyanskaya, Aaron Wong
Providence Medical Research Center, Providence Health Care, Spokane, WA: Katherine Tuttle
Stanford University, Palo Alto, CA: Dejan Dobi, Yury Goltsev
SUNY Buffalo, Buffalo, NY: Brandon Ginley, Nicholas Lucarelli, Brendon Lutnick, Pinaki Sarder
University of California San Diego, La Jolla, CA: Blue Lake, Kun Zhang
University of California San Francisco, San Francisco, CA: Patrick Boada, Maria Joanes, Zoltan Laszik, Andrew Schroeder, Minnie Sarwal, Tara Sigdel, Gary Nolan, Kavya Anjani, Tariq Mukatash
University of Cincinnati, Cincinnati, OH: Rita R. Alloway, Ashley R. Burg, Paul J. Lee, Adele Rike, Tiffany Shi, E. Steve Woodle
University of Michigan, Ann Arbor, MI: Heather Ascani, Ulysses Balis, Victoria Blanc, Ninive C. Conser, Renee Frey, Yougqun He, Oliver He, Jeffrey Hodgin, Matthias Kretzler, Jinghui Luo, Laura Mariani, Rajasree Menon, Edgar Otto, Jennifer Schaub, Becky Steck, Chrysta Lienczewski, Sean Eddy
University of Pittsburgh, Pittsburgh, PA: Michele Elder, Matthew Gilliam, Daniel Hall, John Kellum, Mary Kruth, Raghav Murugan, Paul Palevsky, Parmjeet Randhawa, Matthew Rosengart, Sunny Sims-Lucas, Mary

Stefanick, Stacy Stull, Mitchell Tublin, Tina Vita, James Winters
University of Washington, Seattle, WA: Charles Alpers, Ian de Boer, Ashley Berglund, Ashveena Dighe, Jonathan Himmelfarb, Robyn Mcclelland, Sean Mooney, Stuart Shankland, Kayleen Williams, Kristina Blank, Jonas Carson, Frederick Dowd, Zach Drager, Christopher Park, Stephanie M. Grewenow, Andrew N. Hoofnagle, Chistine Limonte, Kasra Rezaei, James Snyder, Ruikang Wang, Adam Wilcox
UT Health San Antonio, Center for Renal Precision Medicine, San Antonio, TX: Kumar Sharma, Guanshi Zhang, Shweta Bansal, Manjeri Venkatachalam, Richard Montellano, Annapurna Pamreddy, Hongping Ye
UT Southwestern Medical Center, Dallas, TX: S. Susan Hedayati, Asra Kermani, Simon Lee, Christopher Lu, Tyler Miller, Orson Moe, Jiten Patel, Anil Pillai, Harold Park, Kamalanathan Sambandam, Francisco Sanchez, Jose Torrealba, Robert Toto, Miguel Vazquez, Nancy Wang, Dianbo Zhang
Vanderbilt University, Nashville, TN: Richard M Caprioli, Nathan Patterson, Kavya Sharman, Jevvrey M. Spraggins, Raf Van de Plas
Washington University in St. Louis, St. Louis, MO: Jeanine Basta, Sabine M. Diettman, Joe Gaut, Sanjay Jain, Anitha Vijayan, Michael I. Rauchman
Yale University, New Haven, CT: Tanima Arora, Lloyd Cantley, Vijaykumar R. Kakade, Melissa Shaw, Randy Luciano, Dennis Moledina, Ugwuowo Ugochukwu, Francis Perry Wilson, Sandy Alfano

For the TRIBE-AKI Consortium
Johns Hopkins University School of Medicine, Baltimore, Maryland: Steven Menez, Heather Thiessen-Philbrook, Yaqi Jia, Wassim Obeid, Chirag Parikh
Yale School of Medicine, New Haven, Connecticut: Dennis G. Moledina, Sherry G. Mansour,
ICES, Ontario, Canada: Eric McArthur
University of Chicago, Chicago, Illinois: Jay L. Koyner
University of California San Francisco, San Francisco, California: Michael Shlipak
Icahn School of Medicine at Mount Sinai, New York, New York: Steven G. Coca
Schulich School of Medicine and Dentistry, Western University, London, Ontario, Canada: Amit X. Garg
