# Supplementary material for: Urinary EGF and MCP-1 and risk of CKD after cardiac surgery
Source: JCI Insight. 2021 Jun 8;6(11):e147464. doi: 10.1172/jci.insight.147464 (PMC8262289; doi:10.1172/jci.insight.147464)
Supplement: Trial reporting checklists [file jciinsight-6-147464-s255.pdf]

CHECKLIST FOR REVISED SUBMISSION

REQUIRED FILES

_X_PDF of the revised manuscript that includes a preface of point-by-point responses to reviewer comments.

_X_Text document (formatted as Word or RTF) of the revised manuscript (do not include point-by-point responses).

_N/A_PDF of the uncropped/unedited versions of all gel and blot images that appear in the manuscript and Supplement. Each image should be annotated as "Full unedited gel for Figure X"; highlight which lanes of the unedited gel correspond to those shown in the cropped images within the figures.

_X_ Conflict of interest documentation for all authors and appropriate flow diagrams and checklists as required for the Clinical Medicine category (https://nam02.safelinks.protection.outlook.com/?url=https%3A%2F%2Finsight.jci.org%2Fkiosks%2Fauthors%23CMED_Requirements&data=04%7C01%7Csmenez1%40jhmi.edu%7Cb7c2345048104f4994bd08d8dea1fbc7%7C9fa4f438b1e6473b803f86f8aedf0dec%7C0%7C0%7C637504134581254158%7CUnknown%7CTWFpbGZsb3d8eyJWIjoiMC4wLjAwMDAiLCJQIjoiV2luMzIiLCJBTiI6Ik1haWwiLCJXVCI6Mn0%3D%7C1000&sdata=medyKfhUjGhwN7ubZJ0Qgn5tdd%2Bn7Liwnz4FYkdblAw%3D&reserved=0

_X_Provide high-resolution electronic files for all figures. See figure instructions for more information:
https://nam02.safelinks.protection.outlook.com/?url=https%3A%2F%2Finsight.jci.org%2Fkiosks%2Fpublish%2Ffigures&data=04%7C01%7Csmenez1%40jhmi.edu%7Cb7c2345048104f4994bd08d8dea1fbc7%7C9fa4f438b1e6473b803f86f8aedf0dec%7C0%7C0%7C637504134581254158%7CUnknown%7CTWFpbGZsb3d8eyJWIjoiMC4wLjAwMDAiLCJQIjoiV2luMzIiLCJBTiI6Ik1haWwiLCJXVCI6Mn0%3D%7C1000&sdata=NmG%2BDWxqR4o4RDVSCJHIidA1dpVUXEOCxrdbQh%2B6vbA%3D&reserved=0

_X_Provide any supplemental material referenced in the manuscript as (a) (if applicable) a PDF highlighting reviewer-requested changes; and (b) a clean, publication-quality PDF. Other acceptable formats are spreadsheets and videos.

RECOMMENDED

_X_A graphical abstract -- a single schematic image that visually represents the primary findings of an article, allowing readers to easily identify the article's main message -- is strongly encouraged for all revised manuscripts. For details, see:
https://nam02.safelinks.protection.outlook.com/?url=https%3A%2F%2Finsight.jci.org%2Fkiosks%2Fpublish%2Fgraphical&data=04%7C01%7Csmenez1%40jhmi.edu%7Cb7c2345048104f4994bd08d8dea1fbc7%7C9fa4f438b1e6473b803f86f8aedf0dec%7C0%7C0%7C637504134581254158%7CUnknown%7CTWFpbGZsb3d8eyJWIjoiMC4wLjAwMDAiLCJQIjoiV2luMzIiLCJBTiI6Ik1haWwiLCJXVCI6Mn0%3D%7C1000&sdata=Pktrwd18a%2BgpSX7%2BLDgcKlKYImrUVQeSRvXOWuKmeC0%3D&reserved=0

DATA REPORTING GUIDELINES
N/A The exact number of samples (n) for each figure panel representing multiple experiments is included in the legend.

N/A For representative experiments, the number of times the experiment was conducted is stated.

_N/A_ The statistical test utilized is described in each figure legend.

_X_ Error bars are defined, either in Statistics or in the legends themselves; e.g., "Data represent mean ± SEM." Variance around the mean and statistical analysis should not be presented if fewer than 3 independent samples are included.

_X_ For statistical analysis, the analysis appropriately corrects for multiple comparisons when comparing more than two groups and corrects for repeated measures when comparing multiple measurements within subjects. A statement describing inclusion/exclusion criteria is included if samples were excluded from the analysis.

_X_ Graphs of quantitative data must be presented in a manner that clearly shows the distribution of data and variation. Graphs should be presented as dot plots, with the average and appropriate error bars indicated; or as box and whisker plots, with values defined in the legend (the bounds of the boxes, the lines within the boxes, the whiskers, and any outlying values). Presenting data as columns with error bars (dynamite plunger plots) is not permitted.

N/A If lanes in a gel or blot image of any kind are spliced together into a composite image, they must be separated by a thin vertical dividing line, and a note must be included in the legend that the lanes were run on the same gel but were noncontiguous. A black dividing should be used for images with a grey background; a white dividing line should be used for images with a black background.

N/A Histological panels and insets: scale bars included in the images are defined in the figure legends, or original magnification is provided in the figure legends.

N/A For animal models, the precise genotype, strain, number of backcrosses, sex, and age of animals studied are reported.

N/A A description of antibodies utilized is included in Methods, including the source and catalog/clone number for commercial antibodies or a description/reference to a description of the generation of custom antibodies.

_N/A The source of cell lines utilized is indicated.

N/A Large datasets for gene expression microarrays, SNP arrays, and high-throughput sequencing studies are deposited in a public repository. The accession number is provided in the text of the main manuscript. Deposition of other types of large datasets in a public repository is strongly encouraged.

MANUSCRIPT FORMATTING (required components)

_X_ Title: The title should be clear, concise, and limited to 15 words, including conjunctions. The relevant disease or disease model studied should be included. Do not use subtitles, colons, periods, or nonstandard abbreviations.

_X_ Authors: full names of all authors are provided in the appropriate order.

_X_ Affiliations: numbered consecutively according to the order in which authors are listed; include complete locations (city, state, country).

_X_ Conflict of interest statement: declare all author conflicts per the conflict-of-interest policy (https://nam02.safelinks.protection.outlook.com/?url=https%3A%2F%2Finsight.jci.org%2Fkiosks%2Fauthors%23Editorial_Policies&data=04%7C01%7Csmenez1%40jhmi.edu%7Cb7c2345048104f4994bd08d8dea1fbc7%7C9fa4f438b1e6473b803f86f8aedf0dec%7C0%7C0%7C637504134581264153%7CUnknown%7CTWFpbGZsb3d8eyJWIjoiMC4wLjAwMDAiLCJQIjoiV2luMzIiLCJBTiI6Ik1haWwiLCJXVCI6Mn0%3D%7C1000&sdata=tIg2RU2cGq4HVaW1ePkE4yDiCokFAgHcLsoshuOQkck%3D&reserved=0). If no conflict exists, state the following: "The authors have declared that no conflict of interest exists."

_X_ Full mailing address along with telephone and e-mail address of each corresponding author is provided.

MANUSCRIPT TEXT AND REFERENCES

_X_ Text is organized in the following consecutive sections: Abstract, Introduction, Results, Discussion, Methods, Author contributions, Acknowledgments, References, Figure legends, Tables. The recommended text length is 9000 words; text cannot exceed 12000 words, including figure legends, tables, and references.

_X_ Abstract does not exceed 250 words and does not contain primary data or reference citations.

_X_ All nonstandard abbreviations (i.e., abbreviations that do not appear in the Standard abbreviations list; https://nam02.safelinks.protection.outlook.com/?url=https%3A%2F%2Finsight.jci.org%2Fkiosks%2Fpublish%2Fabbreviations&data=04%7C01%7Csmenez1%40jhmi.edu%7Cb7c2345048104f4994bd08d8dea1fbc7%7C9fa4f438b1e6473b803f86f8aedf0dec%7C0%7C0%7C637504134581264153%7CUnknown%7CTWFpbGZsb3d8eyJWIjoiMC4wLjAwMDAiLCJQIjoiV2luMzIiLCJBTiI6Ik1haWwiLCJXVCI6Mn0%3D%7C1000&sdata=W9X%2BsoPmWUZ1Lz1PMyfgraoD3gt7V0YM9pzI3Ua9mjs%3D&reserved=0) are defined at first use in the abstract and again at first use in the text.

_X_ All gene symbols are consistent with Entrez Gene nomenclature, are italicized, and are capitalized according to biological species. See Gene Nomenclature and Style for details: https://nam02.safelinks.protection.outlook.com/?url=https%3A%2F%2Finsight.jci.org%2Fkiosks%2Fpublish%2Fgenestyle&data=04%7C01%7Csmenez1%40jhmi.edu%7Cb7c2345048104f4994bd08d8dea1fbc7%7C9fa4f438b1e6473b803f86f8aedf0dec%7C0%7C0%7C637504134581264153%7CUnknown%7CTWFpbGZsb3d8eyJWIjoiMC4wLjAwMDAiLCJQIjoiV2luMzIiLCJBTiI6Ik1haWwiLCJXVCI6Mn0%3D%7C1000&sdata=ygUXL%2F0%2BNsK3UPWDBkwkO0mXVaWtiOISCIh%2BhMbGwaY%3D&reserved=0

_X_A section at the end of Methods titled "Statistics" summarizes methods of statistical analysis, including the P value that was considered significant.

_X_A brief paragraph following the statistics section titled "Study approval" provides, as appropriate, statements indicating approval of animal and/or human studies, with the official name and location of the relevant review board(s) specified; for human studies, a statement indicating receipt of written informed consent from participants or their guardians is included. For manuscripts that include patient photographs, a separate statement must be included that written informed consent was provided for pictures appearing in the manuscript.

_X_A paragraph following Methods titled "Author contributions" specifies how each author (identified by initials) contributed to the manuscript. Examples include designing research studies, conducting experiments, acquiring data, analyzing data, providing reagents, and writing the manuscript. Multiple contributions may be listed for a single individual, and more than one individual may be associated with a single contribution. For manuscripts with 2 or more co–first authors, the method used in assigning the authorship order among co–first authors must be stated.

_X_Unpublished data, manuscripts in preparation or under review, and personal communications are cited in the text as follows: "([author's name], unpublished observations)." Authors citing the unpublished observations of someone outside of their research team must submit written permission to cite the unpublished results.

_X_References are numbered consecutively according to the order in which they are first cited in the text and are cited in parentheses, e.g., "(1, 2)" or "(4–10)."

_X_Manuscripts accepted for publication or published online are treated as numbered references.

_X_Figures and tables are cited in numerical order; the words "Figure" and "Table" are spelled out.

_X_Complete manufacturer names are provided for each proprietary item used in experiments.

_X_References are styled according to the Instructions for Authors (https://nam02.safelinks.protection.outlook.com/?url=https%3A%2F%2Finsight.jci.org%2Fkiosks%2Fauthors&data=04%7C01%7Csmenez1%40jhmi.edu%7Cb7c2345048104f4994bd08d8dea1fbc7%7C9fa4f438b1e6473b803f86f8aedf0dec%7C0%7C0%7C637504134581264153%7CUnknown%7CTWFpbGZsb3d8eyJWIjoiMC4wLjAwMDAiLCJQIjoiV2luMzIiLCJBTiI6Ik1haWwiLCJXVCI6Mn0%3D%7C1000&sdata=9wVgLRq8UkQEz%2BJ027sxVFpCGJSOpUfAFFhOC9jw%2Bzw%3D&reserved=0), with journal titles styled per PubMed.

_X_Terms such as in vivo, in vitro, etc., are not italicized.

FIGURES, LEGENDS AND TABLES

_X_Figure parts are labeled with capital letters: A, B, C, etc. Subparts within figures (e.g., Figure 1B cannot have parts I and II) are not permitted.

_X_Figure legends do not exceed 300 words.

__X__Usage of terms within the figure legends is consistent with that in the figures themselves; the use of abbreviations is consistent throughout the figures and legends.

__X__Tables are included with the submitted Word document and presented in Microsoft Word's table format (i.e., not pasted as an image from another application).

__X__ Column headings in tables apply to all values throughout the column; a new row of column headings may not be used midway through a table.
